# RNA is a critical element for the sizing and the composition of phase-separated RNA–protein condensates

**DOI:** 10.1038/s41467-019-11241-6

**Published:** 2019-07-19

**Authors:** Marina Garcia-Jove Navarro, Shunnichi Kashida, Racha Chouaib, Sylvie Souquere, Gérard Pierron, Dominique Weil, Zoher Gueroui

**Affiliations:** 1 PASTEUR, Department of Chemistry, École Normale Supérieure, PSL University, Sorbonne Université, CNRS, 75005 Paris, France; 2 Sorbonne Université, CNRS, Institut de Biologie Paris-Seine (IBPS), Laboratoire de Biologie du Développement, F-75005 Paris, France; 3 CNRS UMR-9196, Institut Gustave Roussy, F-94800 Villejuif, France; 4 School of Arts and Sciences, Lebanese International University (LIU), Beirut, Lebanon; 5 Faculty of Sciences, Lebanese University, Beirut, Lebanon

**Keywords:** RNA, Biopolymers in vivo, Cell biology, Organelles

## Abstract

Liquid–liquid phase separation is thought to be a key organizing principle in eukaryotic cells to generate highly concentrated dynamic assemblies, such as the RNP granules. Numerous in vitro approaches have validated this model, yet a missing aspect is to take into consideration the complex molecular mixture and promiscuous interactions found in vivo. Here we report the versatile scaffold ArtiG to generate concentration-dependent RNA–protein condensates within living cells, as a bottom-up approach to study the impact of co-segregated endogenous components on phase separation. We demonstrate that intracellular RNA seeds the nucleation of the condensates, as it provides molecular cues to locally coordinate the formation of endogenous high-order RNP assemblies. Interestingly, the co-segregation of intracellular components ultimately impacts the size of the phase-separated condensates. Thus, RNA arises as an architectural element that can influence the composition and the morphological outcome of the condensate phases in an intracellular context.

## Abstract (web summary)

RNA promotes liquid-liquid phase separation and contributes to the ribonucleoprotein granule assembly. Here the authors employ the protein scaffold ArtiGranule and show the role of intracellular RNA in nucleation and sizing of the phase separated condensates.

## Introduction

Membrane-less organelles, by localizing and regulating complex biochemical reactions, are ubiquitous functional subunits of intracellular organization. Among them, ribonucleoprotein (RNP) granules, which include processing bodies (P-bodies), stress granules (SGs), germ granules, nucleoli, Cajal bodies, etc., are supramolecular assemblies of RNA molecules and proteins found in eukaryotic cells^1^. They regulate RNA processing and thereby play a pivotal role in overall gene expression output, whereas their dysfunction is linked to viral infection, cancer, and neurodegenerative diseases^1,2^. Although RNP granules exhibit different compositions and functions depending on the cellular context, they have strikingly common features concerning their biophysical behavior and assembly process. RNP granules behave as highly concentrated liquid-like condensates that rapidly exchange components with the surrounding medium^3,4^ and their formation relies on a self-assembly process^5,6^. A general model has emerged where RNP granules generate from liquid–liquid phase separation, driven by low-affinity interactions of multivalent proteins and/or proteins containing intrinsically disordered regions (IDRs)^7–11^. However, new roles of RNA in the regulation of granule formation, which has been long assigned to protein components, are being uncovered^12^. RNAs can promote phase separation synergistically with protein–protein interactions but also independently^13,14^. In vitro studies identified that RNA modulates the biophysical properties of liquid droplets, by tuning their viscosity and their dynamics^15,16^. By acting as a molecular seed, RNA contributes to the spatiotemporal regulation of phase-separated granules^17,18^. For instance, the polar positioning of germ granules found in *Caenorhabditis elegans* reflects an RNA competition mechanism that regulates local phase separation^19,20^, while rRNA transcription allows the cells to overcome the inherent stochastic nature of phase separation by timely seeding of nucleolus assembly in *Drosophila melanogaster* embryos^21,22^. More recently, it has been proposed that defects in nuclear RNA levels lead to excessive phase separation of IDR-containing RNA-binding proteins (RBPs) such as FUS and TDP-43^23^. Moreover, RNA appears to determine the specificity of the molecular composition of the granules as shown for polyQ-dependent RNA–protein assemblies^24^. In this context, the development of generic methods, integrating the knowledge accumulated from phase separation in vitro studies, would be particularly acute to elucidate the general principles of the structuring role of RNA within a condensed phase in the cellular environment.

In the present study, we describe the ArtiGranule (ArtiG) bottom-up approach to form, within living cells, RNA–protein assemblies that recapitulate the features of phase-separated liquid condensates. First, through genetic engineering, we modified the oligomeric ferritin protein by adding a structured interaction domain, F36M-FKBP, to generate a modular and versatile scaffold capable of self-interacting with low affinity. Upon reaching a critical concentration, this scaffold assembles into micrometer-sized ArtiG condensates within the cytoplasm of the cells. Then we built up the design with a canonical RNA-binding domain to enable the biochemically neutral condensates to recruit endogenous RNAs. We implemented the PUM.HD domain of human Pumilio 1, a translational repressor that accumulates in P-bodies^25^, and we demonstrated that the resulting ArtiG^PUM^ specifically recruit endogenous Pumilio 1 RNA targets. Finally, this method enabled us to uncover the impact of intracellular RNA in different aspects of the condensate assembly: (i) ArtiG^PUM^ form more efficiently than control ArtiG, underlining that the recruitment of endogenous RNAs seeds and facilitates the condensate nucleation. (ii) The size and polydispersity of ArtiG^PUM^ per cell is strikingly reduced, while their number is higher, compared with control ArtiG. This indicates that the incorporation of endogenous RNAs modulates the morphological outcome of phase-separated condensates. (iii) Micrometric bodies composed of P-body components localize at the periphery of ArtiG^PUM^, revealing that ArtiG^PUM^ subsequently and specifically co-segregate the RBPs associated to the Pumilio-targeted RNAs. Reflecting the specificity of these assemblies, the ArtiG^PUM^ do not interact with SG elements, except in response to stress. We suggest that the multivalent RNAs displayed on ArtiG^PUM^ surface act as molecular cues that seed the recruitment of specific subsets of RBPs/RNAs and coordinate the coexistence of endogenous higher-order assemblies, such as P-Body-like and SG-like assemblies. Furthermore, the docking of biochemically different phases, which is a conserved feature of numerous RNP granules, emerges as a parameter that can regulate the size of the condensates by limiting the growth by structural component addition or coalescence.

## Results

### ArtiG assemble by a concentration-dependent phase transition

**Fig. 1:**
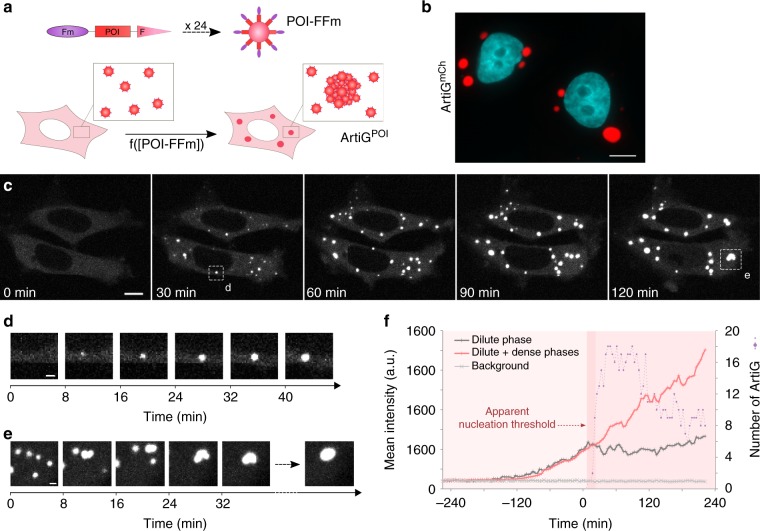
FFm scaffold forms concentration-dependent condensates in living cells. **a** Schematic of POI-FFm multivalent self-interacting scaffold. Genetically engineered ferritins assemble into ArtiG^POI^ through a phase separation process (Fm = F36M-FKBP, POI = protein of interest, F = Ferritin, ArtiG = ArtiGranule). **b** Representative confocal image of ArtiG^mCh^ (red) in HeLa cells, 24 h after transfection of mCherry-FFm construct. Nuclei were stained with Hoechst (blue). Scale bar, 10 µm. **c** Representative time-lapse confocal images of ArtiG^mCh^ (white) nucleation and growth in HeLa cells expressing mCherry-FFm construct. Scale bar, 10 µm. **d** Representative time-lapse confocal images of the growth of an isolated condensate (white). Scale bar, 2 µm. **e** Representative time-lapse confocal images of several ArtiG^mCh^ (white) coalescing into a larger condensate. Scale bar, 2 µm. **f** Comparison of the temporal evolution of the dilute cytosolic mCherry-FFm fluorescence (dilute phase), with the total cytoplasmic fluorescence (dilute + dense phases) for the lower cell shown in **c**. The violet dots represent the number of granules measured as a function of time. For the purpose of representation, the images in **c**, **d**, and **e** are slightly saturated

The formation of RNA–protein condensates is thought to be driven by liquid–liquid phase separation through weak, multivalent interactions between biomolecules^8^. According to this model, the multivalent interactions promote the demixing, whereas their low affinity enables the components to dynamically exchange within the condensed phase and with the surrounding medium. Inspired by these findings, we took advantage of ferritin self-assembly in a 24-mer nanocage to generate a multivalent three-dimensional scaffold, to which we added an interaction module. We engineered the light chain of human ferritin by fusing the F36M-FKBP (Fm) protein to the N terminus of the ferritin monomer. The Fm protein is a point mutant of human FK506-binding protein (FKBP) protein that has the property of forming homodimers with micromolar affinity^26^. The resulting chimeric Fm-ferritin (FFm) has 24 Fm proteins pointing outside the nanocage, which act as self-interacting domains. Thus, every FFm could undergo up to 24 interactions with other FFm nanocages with micromolar affinity. Such a protein scaffold can easily be functionalized by fusing a protein of interest (POI-FFm), for instance a fluorescent protein or a RNA-binding domain (Fig. 1a).

To test the strategy, we first expressed an mCherry-FFm construct in HeLa cells. Twenty-four hours after transfection, about 30% of the transfected cells displayed a few micrometric fluorescent condensates dispersed throughout their cytoplasm, whereas the other cells exhibited homogeneous diffuse cytosolic mCherry-FFm fluorescence (Fig. 1b and Supplementary Fig. 1a). Neither fluorescence nor mCherry-FFm condensates were detected in the nucleus, in agreement with the diameter of the folded mCherry-FFm nanocages, which exceeds the size limit to passively diffuse through nuclear pore complexes. When Fm protein was replaced by the wild-type FKBP protein, which is unable to homodimerize, the resulting FKBP-mCherry-ferritin construct did not promote condensate formation and remained diluted in the cytoplasm of cells (Supplementary Fig. 1b). The formation of FFm condensates was recapitulated in HEK-293 cells with equivalent results (Supplementary Fig. 1c). From now on, the mCherry-FFm condensates will be referred as ArtiG^mCh^ and, more generally, any POI-FFm condensate as ArtiG^POI^ (Fig. 1a).

In order to characterize ArtiG^mCh^ nucleation and growth steps, we next followed mCherry-FFm expression after 8–10 h of transfection using time-lapse live imaging. At low expression levels, mCherry-FFm was diffuse in the cytoplasm of HeLa cells (Fig. 1c, first panel). As mCherry-FFm concentration increased as a function of time, the nucleation of several micrometric fluorescent ArtiG^mCh^ condensates was observed (Fig. 1c). The spherical ArtiG^mCh^ were homogeneously distributed and mobile within the cytoplasm (Supplementary Movie 1). Individual condensates first exhibited a rapid growth phase both in size and fluorescence intensity, before slowing down after about 40–60 min (Fig. 1d and Supplementary Fig. 1d), whereas condensates that encountered one another coalesced to form larger spherical bodies (Fig. 1e and Supplementary Movie 2). We monitored the number of ArtiG^mCh^, as well as the accumulation of the dilute cytosolic mCherry-FFm fluorescence (dilute phase) and of the total cytoplasmic fluorescence (dilute + condensed phases), as a function of time (Fig. 1f and Supplementary Fig. 1e). ArtiG^mCh^ formation occurred within a narrow time window of about 20–30 min; no new granule appearance was observed thereafter. After the burst of granule formation, the number of ArtiG^mCh^ gradually decreased as they underwent fusion. Concurrently with the nucleation of the first ArtiG^mCh^, the cytosolic fluorescence intensity (dilute phase) stopped increasing to eventually reach a stationary phase, whereas the total cytoplasmic fluorescence intensity (dilute + condensed phases) kept increasing over time. These results suggest that the nucleation of ArtiG^mCh^ occurs at a critical concentration of cytosolic mCherry-FFm, referred to as apparent nucleation threshold in the Fig. 1e and Supplementary Fig. 1e, which is a signature of first-order phase transitions. Furthermore, ArtiG^mCh^ seem to establish a dynamic equilibrium buffering the variations of FFm concentration in the cytoplasm.

Taken together, these data illustrate that FFm scaffold undergoes a phase transition to form ArtiG micrometric condensates within the cytoplasm of living cells, in a concentration-dependent manner.

### ArtiG recapitulate the main properties of liquid droplets

A number of studies has highlighted that membrane-less organelles have specific material properties and behave as liquid or gel compartments^27^. Therefore, we further assessed the morphological and biophysical characteristics of the ArtiG.

**Fig. 2:**
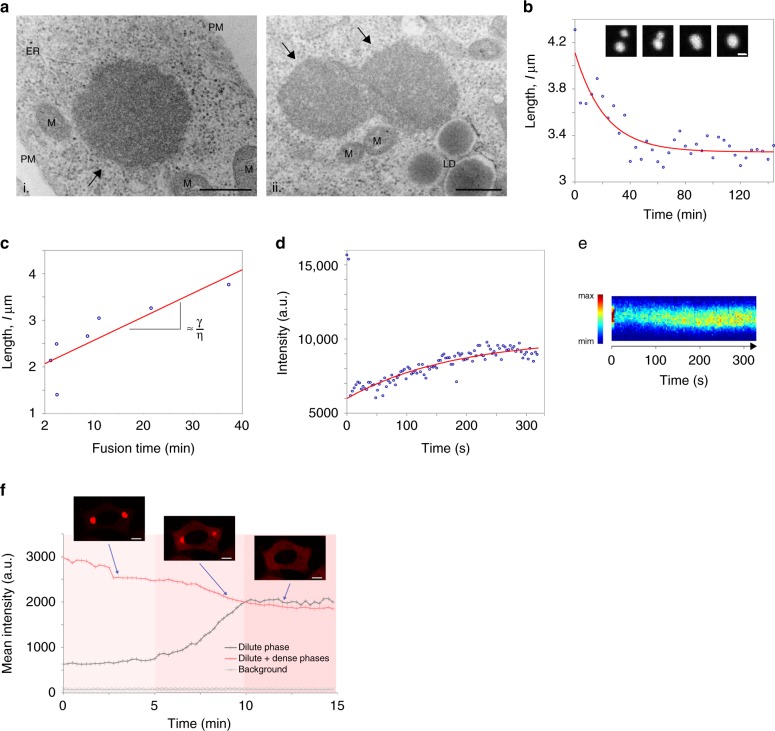
ArtiG^mCh^ recapitulate the main properties of phase-separated liquid droplets. **a** Ultrastructure of an individual ArtiG^mCh^ (i, black arrow) and of two ArtiG^mCh^ (ii, black arrows) undergoing fusion in HeLa cells, 24 h after transfection of mCherry-FFm construct. ER endoplasmic reticulum, M mitochondrion, LD lipid droplet, PM plasma membrane. Scale bar, 500 nm. **b** Temporal evolution of the distance between two ArtiG^mCh^ fusing with each other and relaxing into a single droplet within a characteristic time of 40 min. Insert: time-lapse of the corresponding fusion event. Scale bar, 2 µm. **c** Plots of the characteristic relaxation time of different fusion events as a function of the diameter, with the slope giving the capillary velocity (ratio between the surface tension *γ* and the viscosity *η*). **d** Example of recovery of fluorescence intensity after photobleaching of a 2 µm ArtiG^mCh^. **e** Kymograph representation of the fluorescence recovery of the ArtiG^mCh^ analyzed in **d**. **f** Dissolution of ArtiG^mCh^ upon addition of 2.5 µM of FK506 to the culture medium. Comparison of the temporal evolution of the total cytoplasmic fluorescence (dilute and dense phases) with the dilute cytosolic mCherry-FFm fluorescence (dilute phase) as a function of time

The ultrastructural characterization of ArtiG^mCh^, by electron microscopy of glutaraldehyde-fixed and Epon-embedded thin sections of the mCherry-FFm expressing cells, revealed micrometric round bodies (Fig. 2a). They were not delineated by a membrane and they were usually distant from cytoplasmic organelles such as mitochondria or the endoplasmic reticulum. In agreement with these observations, no fluorescent lipophilic staining was detected around ArtiG^EGFP^ in fluorescence microscopy (Supplementary Fig. 2a) and the different fluorescent ArtiG exhibited generally spherical shapes (Fig. 1 and Fig. 2). Besides, ArtiG that deformed under mechanical stress relaxed back in a spherical shape (e.g., after being trapped between the nuclear and plasma membranes; Supplementary Fig. 2b). These dynamics and the spherical morphology indicate a liquid–liquid demixing state of the ArtiG.

An additional hallmark of phase separation displayed by ArtiG is their tendency to fuse. As observed in Fig. 1e and Fig. 2b, nearby ArtiG^mCh^ tend to coalescence to form larger bodies, which could give rise to cells displaying a few large spherical condensates 24 h after transfection (Fig. 1b and Supplementary Fig. 1a). By computing the fusion time as a function of the diameter of the resulting ArtiG^mCh^ (Fig. 2c) and by assuming that surface tension drives the fusion process whereas viscosity tends to impede it, we determined that ArtiG^mCh^ have an effective viscosity of about 10^3^ Pa.s (Methods). This is in the same order of magnitude as the effective viscosity measured for endogenous condensates such as nucleoli in *Xenopus laevis*, large germ granules in *C. elegans* oocytes, and nuclear speckles in CHO cells^4,28,29^.

We next performed fluorescence recovery after photobleaching experiments (FRAP) and found that, after photobleaching of the ArtiG^mCh^ components, a fraction of the fluorescent signal (35%) recovered with a timescale of about 2 min for micrometric granules (Fig. 2d, e). This illustrates that ArtiG^mCh^ are stable structures in which part of the components are mobile and exchange with the surrounding cytoplasm.

To further examine ArtiG^mCh^ stability, we tested their resistance to dilution and to chemical dissociation of Fm–Fm homodimers. When diluted in the lysis buffer, ArtiG^mCh^ retained the same morphology as observed in living cells, even after an incubation of 16 h at room temperature (RT; Supplementary Fig. 2c). In contrast, micrometric ArtiG^mCh^ tend to disappear within few minutes upon addition of FK506, a specific competitor of the Fm–Fm interaction^26^, to the cell culture medium. This demonstrates that Fm–Fm interactions are essential to maintain the integrity of the condensates. Concomitantly to ArtiG^mCh^ dissolution, a large increase of the cytosolic fluorescence was observed, indicating that ArtiG are comparable to protein reservoirs (Fig. 2f and Supplementary Fig. 2d).

Taken together, these data demonstrate that ArtiG^mCh^ recapitulate the hallmarks of liquid-like behavior described for native RNP granules, as they are spherical and relax into one spherical droplet after fusion and deformation, but also as they dynamically exchange components with the dilute cytosolic phase. Moreover, despite their sensibility to Fm–Fm homodimer disruption, ArtiG^mCh^ resist dilution as seen for many cellular condensates, illustrating that stable assemblies can form from weak interactions.

### Engineered ArtiG^PUM^ recruit a specific endogenous RNA subset

To shed light on the impact of RNA on phase separation of RNA–protein condensates in a native cellular environment, we implemented a canonical RNA-binding domain in our multivalent FFm scaffold. We chose the Pumilio homology domain (PUM.HD) of human Pumilio 1 protein as follows: (i) PUM.HD is a well-characterized RNA-binding domain^30^; (ii) Pumilio 1 is known to bind specific RNA elements that accumulate in P-bodies^25^, but do not promote their assembly when overexpressed in cells^31^; (iii) by its RNA-binding activity, Pumilio 1 is a key regulator of numerous cellular processes, including translation repression^32^. To generate PUM.HD-FFm self-interacting multivalent scaffold, PUM.HD was inserted between the Fm protein and the ferritin monomer (Fig. 1a).

**Fig. 3:**
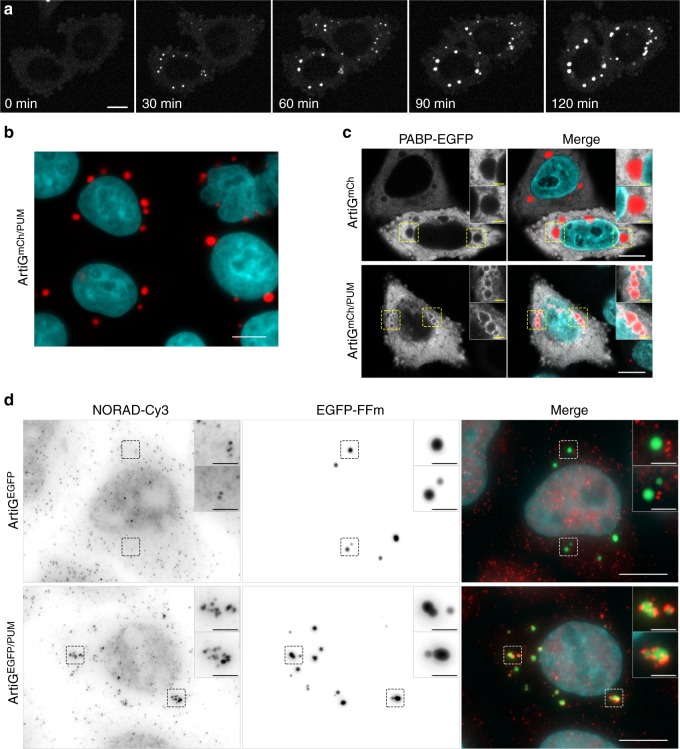
ArtiG^PUM^ recruit specific endogenous RNAs. **a** Representative time-lapse confocal images of HeLa cells expressing mCherry-FFm and PUM.HD-FFm constructs. Scale bar, 10 µm. For the purpose of representation, the images are slightly saturated. **b** Representative confocal image of ArtiG^mCh/PUM^ (red) in HeLa cells, 24 h after transfection of mCherry-FFm and PUM.HD-FFm constructs. Nuclei were stained with Hoechst (blue). Scale bar, 10 µm. **c** To visualize polyadenylated RNA, a PABP-EGFP fusion (white) was co-transfected with mCherry-FFm and PUM.HD-FFm (red) into HeLa cells 24 h before fixation. Confocal imaging. Scale bar, 10 µm. Zoom, 2 µm. **d** Epifluorescence imaging of NORAD lncRNA following smFISH-Cy3 (red in merge) in HeLa cells expressing ArtiG^EGFP^ (green in merge, upper row) and ArtiG^EGFP/PUM^ (green in merge, lower row). Nuclei were stained with DAPI (blue). Scale bar, 10 µm. Zoom, 2 µm

We first confirmed by co-expressing EGFP-FFm and mCherry-FFm constructs that cells could display ArtiG^EGFP/mCh^ hybrid condensates containing two different ferritins (Supplementary Fig. 3a). We next directed the formation of hybrid ArtiG^mCh/PUM^ by co-expressing mCherry-FFm and PUM.HD-FFm, in a 5:1 plasmid ratio, and we monitored the protein expression by western blot analysis (Supplementary Fig. 3b). We assessed the formation of ArtiG^mCh/PUM^ by time-lapse confocal microscopy 8–10 h after transfection (Fig. 3a). As for ArtiG^mCh^, the fluorescence was first diffused in the cytoplasm before concentrating in several bright bodies that grew as a function of time in a concentration-dependent manner (Fig. 3a and Supplementary Fig. 3c). Twenty-four hours after transfection, cells displayed several micrometric condensates (Fig. 3b). On the other hand, when we transfected a FKBP-mCherry-PUM.HD-ferritin construct, which is multimeric but cannot self-interact, no fluorescent condensates were observed (Supplementary Fig. 3d). As shown for ArtiG^mCh^, ArtiG^mCh/PUM^ were stable in cellular lysates, while they were disrupted within few minutes in cells upon addition of FK506 to the culture medium (Supplementary Fig. 3e, f). This behavior has also been observed for the core components of SGs^33^ and for P-bodies in lysates^25^. In order to assess the presence of RNA in ArtiG^mCh/PUM^, we analyzed the localization of an enhanced green fluorescent protein (EGFP)-fused Poly(A)-binding protein (PABP-EGFP) as an indirect fluorescent reporter of polyadenylated RNAs. As shown in Fig. 3c, when overexpressed, PABP-EGFP accumulated around ArtiG^mCh/PUM^, which exhibited an enriched corona of EGFP fluorescent signal, but not around control ArtiG^mCh^. Collectively, the co-expression of an RNA-binding PUM.HD-FFm scaffold with a neutral mCherry-FFm scaffold eventually leads to the concentration-dependent phase separation of micrometric protein condensates, which recruit polyadenylated RNAs.

We then investigated the specificity of RNA recruitment into ArtiG^mCh/PUM^. We focused on NORAD, a highly conserved and abundant long noncoding RNA (lncRNA), which has been described as a multivalent binding-platform of PUM proteins and, by there, a key regulator of their activity^34^. We examined the intracellular localization of endogenous NORAD lncRNA using the recently developed smiFISH (single-molecule inexpensive fluorescence in situ hybridization) technique^35^. We found that ArtiG^EGFP/PUM^ were enriched in NORAD lncRNA (Fig. 3d). Strikingly, NORAD-cy3 FISH signal was localized into micrometer-sized discrete patches on ArtiG^EGFP/PUM^. By contrast, ArtiG^EGFP^ were devoid of any NORAD-cy3 signal (Fig. 3d). Similar results were obtained with two other Pumilio 1 targets, PUM2 and Rad21 mRNAs, which were also recruited by ArtiG^EGFP/PUM^ (Supplementary Fig. 3g). To assess whether RNA recruitment on the ArtiG^EGFP/PUM^ was specific, we performed a smiFISH to detect RAB13 mRNA, which is not a Pumilio 1 target and is known to localize at tips of protrusions^36^. As expected, no colocalization between ArtiG^EGFP/PUM^ and RAB13 mRNA was observed (Supplementary Fig. 3h). Thus, these results show that ArtiG scaffold can be used to specifically target endogenous RNAs within a phase-separated condensate in cells.

### Resulting P-body component co-segregation impacts ArtiG^PUM^

**Fig. 4:**
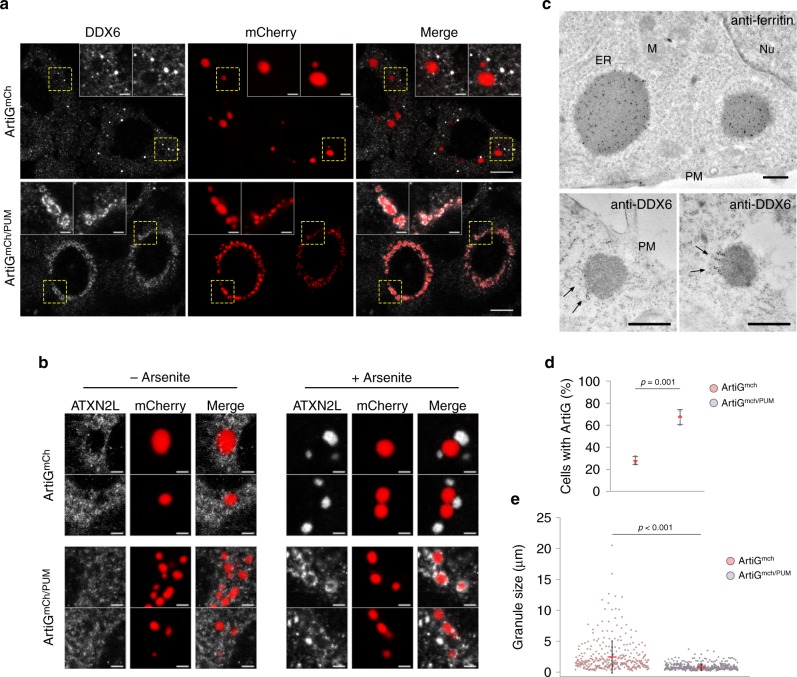
RNA modifies ArtiG^PUM^ composition, nucleation incidence, and morphology. **a** HeLa cells expressing ArtiG^mCh^ and ArtiG^mCh/PUM^ (red) were fixed and analyzed by immunofluorescence using antibodies recognizing endogenous DDX6 (white). Confocal images. Scale bar, 10 µm. Zoom, 2 µm. **b** HeLa cells expressing ArtiG^mCh^ and ArtiG^mCh/PUM^ (red) were treated or not with arsenite for 30 min, fixed, and analyzed by immunofluorescence using antibodies recognizing endogenous ATXN2L (white). Enlarged regions of the confocal images presented in Supplementary Fig. 4c. Scale bar, 2 µm. **c** As indicated, ferritin and endogenous DDX6 were identified in thin sections of ArtiG^mCh/PUM^-expressing cells, using a secondary antibody coupled to 10 nm gold particles. Ferritin is concentrated all-over ArtiG^mCh/PUM^ (upper panel), whereas DDX6 accumulates at the edge of the condensates (lower panels, black arrows). Scale bar, 500 nm. ER endoplasmic reticulum, M  mitochondrion, Nu nucleus, PM plasma membrane. **d** Percentage of transfected cells displaying ArtiG condensates 24 h after transfection of mCherry-FFm and PUM.HD-FFm constructs. Data represent the mean ± SD of three independent experiments. **e** Quantification of the size distribution of ArtiG^mCh^ and ArtiG^mCh/PUM^. Dots represent single measurements of ArtiG size and are pooled from three independent experiments. Means and SDs are superimposed

In a native context, the endogenous RNAs interact with specific RBPs that regulate their biogenesis and their fate. A recent study, unraveling the human P-body proteome and transcriptome, has reported that both Pumilio proteins and their RNA targets are significantly enriched in P-bodies^25^. With this in mind, we assessed the presence of DDX6, EDC4, and LSM14A, as P-body markers, and ATXN2L as SG marker, in ArtiG^mCh/PUM^ by immunostaining 24 h after transfection followed by confocal microscopy. Consistent with the neutral design of the ArtiG^mCh^ condensates, ArtiG^mCh^ did colocalize with neither DDX6-, EDC4-, LSM14A-labeled P-bodies (Fig. 4a and Supplementary Fig. 4a, b), nor ATXN2L, even after arsenite-induced SG formation (Fig. 4b and Supplementary Fig. 4c). In contrast, ArtiG^mCh/PUM^ clearly displayed a DDX6 patchy corona on their surface (Fig. 4a) and a similar result was obtained after EDC4 and LSM14A immunostaining, which formed micrometric patches attached to ArtiG^mCh/PUM^ (Supplementary Fig. 4a, b). This patchy organization around ArtiG was also observed by electron microscopy imaging. The immunogold detection of endogenous DDX6 on thin sections of ArtiG^mCh/PUM^ expressing cells reported the recurrent presence of distinct DDX6-labeled assemblies at the boundary of the condensates (Fig. 4c and Supplementary Fig. 4d). FRAP experiments showed that ArtiG^mCh/PUM^ behave as gel-like bodies, displaying almost no recovery of mCherry fluorescence after photobleaching (Supplementary Fig. 4e). In contrast, patches of GFP-labeled LSM14A around the ArtiG^mCh/PUM^ recovered to up to about 80% within 30 s after photobleaching (Supplementary Fig. 4e). In addition, ATXN2L fluorescent immunostaining of ArtiG^mCh/PUM^-expressing cells revealed that endogenous ATXN2L was mainly diffuse in the cytoplasm in the absence of stress (Fig. 4b and Supplementary Fig. 4c). Nevertheless, when cells were exposed to arsenite to induce bona fide SGs, micrometric ATXN2L patches were localized to ArtiG^mCh/PUM^ surface (Fig. 4b and Supplementary Fig. 4c). Altogether, these data demonstrate that ArtiG^PUM^ selectively co-segregate P-body components in non-stressed cells, whereas SG components dock to ArtiG^PUM^ exclusively after arsenite stress.

As ArtiG^PUM^ directly and indirectly incorporated components of endogenous RNP granules, we hypothesized that these endogenous components may modify the behavior of the synthetic condensates. First, we investigated the nucleation incidence of ArtiG^mCh/PUM^ in comparison with ArtiG^mCh^ 24 h after transfection. ArtiG^mCh/PUM^ could be observed in ~ 70% of transfected cells, whereas ArtiG^mCh^ could only be detected in ~ 30% of transfected cells (Fig. 4d), despite exhibiting similar protein levels (Supplementary Fig. 3b). Remarkably, ArtiG^mCh/PUM^ displayed a smaller size (mean ± SD = 0.83 ± 0.52 µm) and narrower size distribution (Coefficient of Variation, CV = 62 %) than ArtiG^mCh^ (2.44 ± 2.60 µm, CV = 107 %) (Fig. 4e).

Taken together, these results indicate that ArtiG^mCh/PUM^ establish a crosstalk with endogenous RNP components that organize in patchy assemblies around the ArtiG condensed phase. Furthermore, these interactions have a direct impact on the formation of the condensates, in terms of nucleation incidence and size.

### RNA and binding partners regulate condensate size

**Fig. 5:**
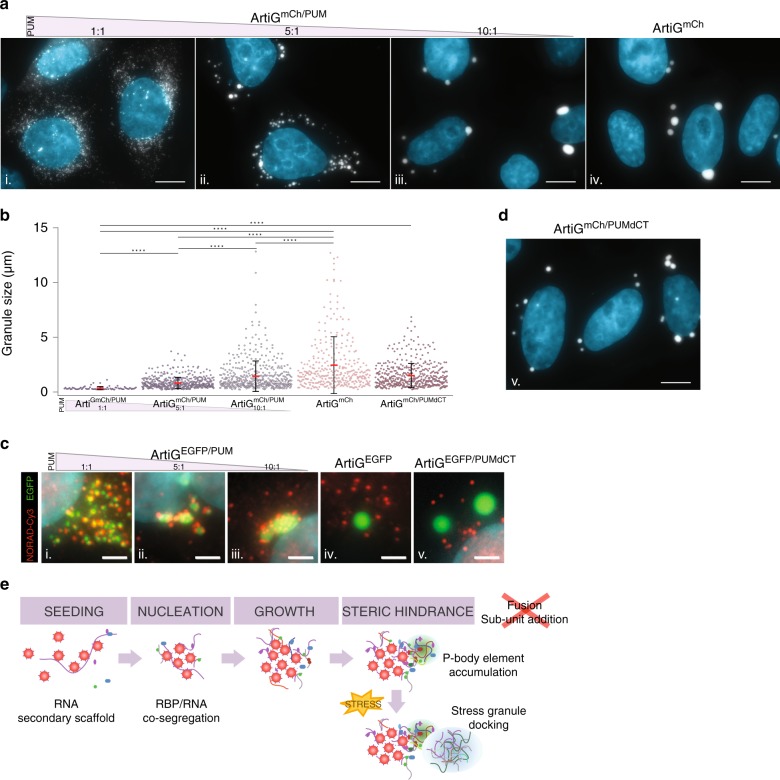
RNA is a critical element for ArtiG^PUM^ size scaling. **a** Representative confocal images of ArtiG^mCh/PUM^ (white) in HeLa cells, 24 h after transfection of mCherry-FFm and PUM.HD-FFm constructs in a plasmid ratio of 1:1 (i), 5:1 (ii), 10:1 (iii), and 1:0 (iv). Nuclei were stained with Hoechst (blue). Scale bar, 10 µm. **b** Quantification of the size distribution of ArtiG^mCh/PUM^ and ArtiG^mCh^ described in **a**. Dots represent single measurements for ArtiG size and are pooled from three independent experiments. Means and SDs are superimposed. ****p < 0.0001. For representation, the two largest ArtiG^mCh^ ( > 15 µm, as shown in Fig. 4e) were not included. **c** Epifluorescence images of NORAD lncRNA following smFISH-Cy3 (red) in HeLa cells expressing ArtiG^EGFP/PUM^, ArtiG^EGFP^, and ArtiG^EGFP/PUMdCT^ (green). The panels show enlarged regions of the cells represented in Supplementary Fig. 5b. Nuclei were stained with DAPI (blue). Scale bar, 2 µm. **d** Representative confocal image of ArtiG^mCh/PUMdCT^ (white) in HeLa cells, 24 h after transfection of mCherry-FFm and PUMdCT-FFm constructs in a plasmid ratio of 1:1. Nuclei were stained with Hoechst (blue). Scale bar, 10 µm. **e** Schematic of the model for ArtiG^PUM^ nucleation, growth, and co-segregation of endogenous RNPs

In order to assess more precisely the contribution of RNA in modifying the morphology and behavior of the composite condensates, we tuned the relative ratio of PUM.HD within the ArtiG. We transfected different plasmid ratios: 1:1, 5:1, and 10:1 of mCherry-FFm and PUM.HD-FFm constructs, and protein level variation was confirmed by western blotting analysis (Supplementary Fig. 5a). Strikingly, fluorescence microscopy image analysis revealed that ArtiG^mCh/PUM^ size and number scaled with the plasmid ratio (Fig. 5a). The more the ArtiG were enriched in RNA-binding elements (PUM.HD-FFm), the more abundant and the smaller they were in the cells (Fig. 5a, b). Regarding conditions with a plasmid ratio of 1:1, cells displayed a large number of diffraction-limited ArtiG^mCh/PUM^, ~ 60 condensates per cell in average (of which 90 % was smaller than 0.4 µm in diameter, the diffraction limit of our optical setup). Electron microscopy acquisitions allowed for the quantification of ArtiG^mCh/PUM^ size and dispersion (0.35 ± 0.15 µm, CV = 42 %) (Fig. 5b). Interestingly, these sub-micrometric ArtiG^mCh/PUM^ coexisted without coalescing into larger condensates, despite their high concentration in the cytoplasm (Fig. 5a, panel i). In contrast, cells transfected with the plasmid ratio of 10:1 had a reduced number of ArtiG^mCh/PUM^ (in average ~ 12 condensates per cell) with a size and a distribution of 1.44 ± 1.39 µm (CV = 97%), comparably to neutral ArtiG^mCh^ (~ 4 ArtiG^mCh/PUM^ per cell in average, 2.44 ± 2.60 µm, CV = 107 %) (Fig. 5a, panels iii and iv, b). In this condition, ArtiG^mCh/PUM^ fused to relax into larger condensates (Supplementary Movie S3). Finally, as described before, the plasmid ratio of 5:1 led to cells exhibiting an intermediate phenotype (~19 ArtiG^mCh/PUM^ per cell in average, with 0.83 ± 0.52 µm in size, CV = 62%) (Fig. 5a, panel ii, b). Single-molecule detection of NORAD lncRNA confirmed that, despite the drastic size changes, all the ArtiG^EGFP/PUM^ (1:1, 5:1, and 10:1) retained the capacity to bind RNA (Fig. 5c and Supplementary Fig. 5). The recruitment of RNA by the condensates was associated to the specific recruitment of P-body elements in patchy micro-assemblies at the surface of ArtiG^mCh/PUM^ (Supplementary Fig. 5c). Even in the most extreme condition of plasmid we tested (ratio of 10:1), we still observed that ArtiG^mCh/PUM^ recruited RNA and P-body elements, indicating that even in minute amounts, the presence of PUM.HD-FFm affected the composition and behavior of the granules. These results suggested that the RNA-binding capacity, and thereby the recruitment of RNA and associated proteins, could directly modulate the morphologic fate of the phase-separated condensates in terms of size and number.

To further validate the leading role of RNA in shaping the condensate phase, we constructed a deletion mutant of PUM.HD-FFm in order to reduce its RNA-binding capacity. PUM.HD is a sequence-specific RNA-binding domain, which is characterized by a tandem of eight imperfect copies of a structural motif (R1 to R8), that respectively interact with a different ribonucleotide of the targeted RNA sequence^30,37^. We hypothesized that by truncating the C-terminal region of PUM.HD (mutant PUMdCT), we could weaken its RNA-binding capacity and hence the sizing phenotype of ArtiG^PUM^. After confirming the assembly of ArtiG^mCh/PUMdCT^ mutant condensates in living cells (Fig. 5d and Supplementary Fig. 6a for western blot analysis), we next assessed that the deletion of the last two motifs, R7 and R8, was sufficient to impair the RNA-binding capacity of PUM.HD. Colocalization experiments reported that ArtiG^mCh/PUMdCT^ were able to recruit neither NORAD lncRNA (Fig. 5c, panel v, and Supplementary Fig. 5b) nor cytoplasmic PABP-EGFP at their surface (Supplementary Fig. 6b). Consistently, ArtiG^mCh/PUMdCT^ did not display any patchy micrometric assembly of the P-body or SG components tested, at their periphery (Supplementary Fig. 6c, d). Finally, in the same transfection conditions (1:1 plasmid ratio with mCherry-FFm, Supplementary Fig. 6a for western blotting analysis), ArtiG^mCh/PUMdCT^ displayed larger size and size distribution than wild-type ArtiG^mCh/PUM^: 1.50 ± 1.11 µm (CV = 74%) vs. 0.35 ± 0.15 µm (CV = 42%) (Fig. 5b). Again, the number of condensates per cell was inversely correlated with size, as cells exhibited ~10 ArtiG^mCh/PUMdCT^ per cell in average. These results demonstrated that the recruitment of RNA and the resulting recruitment of its interacting partners are the critical elements responsible for the morphological changes of the phase-separated ArtiG^PUM^.

Altogether, our data show that by conferring the capacity to recruit intracellular RNAs and their binding partners to an initially inert scaffold that can undergo phase separation, we strongly modify the outcome of the condensates in terms of size and number. This illustrates how, in the context of an intracellular phase separation, RNA is a critical architectural component in addition to its function as a carrier of genetic information.

## Discussion

Many of the biophysical hallmarks of phase separation model for RNP assembly have emerged from in vitro reconstitution experiments^7,8^. Nevertheless, the study of phase-separation processes in regards to molecular crowding, promiscuity, posttranslational modifications, etc., requires the development of novel tools operating within the native intracellular environment^38–42^. Inspired by pioneer experiments on liquid–liquid phase separation of multi-domain macromolecules^8^, we engineered a multivalent self-interacting protein scaffold, with the goal of being biochemically inert regarding the cellular environment. Our approach differs from strategies using protein scaffolds enriched with intrinsically disordered proteins (IDPs), which are very interesting for understanding, for instance, the role, promiscuity, and aging of IDP assemblies, but which add a supplementary level of complexity when working in the native context of cells as they undergo unrestrained homotypic interactions.

Our results show that our ArtiG approach forms micrometric assemblies that recapitulate the features of phase-separated condensates (Fig. 1). Once a critical concentration of protein scaffold is reached in the cytoplasm, ArtiG start nucleating. As the ArtiG assemble, a dynamic equilibrium is established in which the concentration of the surrounding cytosolic phase is maintained constant through a buffering process by the condensed phase. The condensed phase incorporates the amount of scaffold continuously supplied by the cell and consequently grows in size (Fig. 1d, f, Supplementary Fig. 1e, and Supplementary Fig. 3c). This buffering property of phase-separated compartments has been discussed as a possible role for intracellular condensates in maintaining the robustness needed for biochemical reactions, despite biological fluctuations^5^. These hallmarks make our system reliable for testing predictions and studying phase separation in an intracellular environment.

We used ArtiG approach to examine the contribution of RNA elements to the formation of a condensed phase within a living system. We generated a simplified archetypal RBP condensate by incorporating a unique RNA-binding domain, PUM.HD, from human Pumilio 1 protein. Our minimal bio-mimicking system succeeds in assembling concentration-dependent RNA–protein condensates within the cytoplasm of living cells and in specifically recruiting Pumilio 1 RNA targets (Fig. 3). Interestingly, the RNA-binding capacity of ArtiG^mCh/PUM^ is associated with a higher incidence of nucleation for these RNA–protein condensates, compared with purely protein-based ones (ArtiG^mCh^) (Fig. 4d). Recent studies have proposed that RNA can provide spatiotemporal information and acts as a multivalent template that favors granule seeding^16–18,22,24,43^. For instance, by carrying multiple binding sites for PUM proteins, NORAD lncRNA functions as a multivalent binding platform that sequesters a significant fraction of the cytosolic PUM proteins^34^. This type of architecture, which is a common feature of numerous RNAs, would confer the capacity to form locally high-concentration assemblies that trigger the nucleation of a condensed phase on RNA. Altogether, these observations support that RNA can act as key regulator of in vivo phase separation by seeding the process^19,22,23,44^.

An open question is how the biochemical composition of RNP granules is determined and maintained^24^. Our results show that micrometric bodies of specific compositions are localized at the periphery of ArtiG^PUM^. The addition of a RNA-binding domain to the protein scaffold confers the capacity to communicate with intracellular components to the initial biochemically inert ArtiG condensates. One could imagine that by exposing and concentrating RNA locally, the ArtiG^PUM^ may recruit RBPs in a nonspecific manner or nucleate SG formation by RNA mislocalization. In contrast, the RNA recruited by the ArtiG^PUM^ specifically co-segregated P-body components, generating peripheral DDX6-, EDC4-, and LSM14A-enriched bodies (Fig. 4a–c and Supplementary Fig. 4a, b). Two non-exclusive models could be envisioned: either the interaction of P-body components with the RNA occurred before its recruitment into the ArtiG^PUM^ or, through the recruitment of a particular subset of RNAs, ArtiG^PUM^ expose molecular cues that spatially specify the nucleation of P-body components (Fig. 5e). This last picture is coherent with recent studies, suggesting that RNP granules form by combinatory intermolecular RNA–protein, RNA–RNA, and protein–protein interactions^12,45–48^. It is interesting to note that, in our system, two biochemically different phases, ArtiG and P-body-like, which share certain components, can coexist apparently immiscibly, as observed for Cajal bodies with attached B-snurposomes^49^. The local coordination of immiscibly condensed phases is even more striking under stress conditions, where we observed SGs interacting with the ArtiG^PUM^ (Fig. 4b and Supplementary Fig. 4c). Similarly, a physical juxtaposition of SGs and P-bodies is also observed when cells are stressed^50–54^. As is seen for the natural granules, under stress conditions ArtiG^PUM^ may share transient interactions with SGs, physically linking the assemblies and leading to the docking of the two compartments (Fig. 5e).

There is a mounting interest in examining the mechanisms underlying size control of organelles during cell growth and embryonic development^55,56^. Indeed, the size of self-organized structures, such as the meiotic spindle or the centrosomes, scales with the cell dimension^57^. One hypothesis is that size control could be mediated by the limiting supply of structural components found within the cell volume^58–61^. Interestingly, intracellular phase transitions provide additional physical bases with which to understand the dependence of membrane-less organelle assembly, number, and size, to the cellular volume^62,63^. We have been able to compare an archetypal phase separation of inert multivalent scaffolds to the phase separation of multivalent scaffolds that recruit a subset of intracellular components. ArtiG^mCh^ nucleation exhibits a concentration dependence typical of first-order phase transitions: upon reaching a critical concentration, multiple high-concentration assemblies emerge from the cytosolic dilute phase and grow in size, buffering the supply of scaffold subunits (Fig. 1). In a simplified picture, a multi-droplet polydisperse mixture will coarsen by Ostwald ripening to form a single droplet, as the steady-state system tends to favor a few large condensates coexisting with the dilute phase^64^. Yet, our observations suggest that, in an intracellular context, the growth of the ArtiG^mCh^ by coalescence dominates over Ostwald ripening. Ultimately, the first-assembled micrometric condensates will grow and coalesce in 1 or 2 large ArtiG^mCh^. In contrast, a large number of size-limited ArtiG^mCh/PUM^ are capable of coexisting within the cell. The RNA recruitment by the ArtiG^mCh/PUM^ has clear consequences for the growth and the polydispersity of the condensates: ArtiG^mCh/PUM^ growth process stops at some point, depending on the amount of biomolecules co-segregated by the condensates (Fig. 5). Accordingly, the growth-limited phenotype of ArtiG^PUM^ was rescued when wild-type PUM.HD was replaced by a mutant with defective RNA-binding activity. RNA appears therefore to be the biomolecule responsible for this feature. Still, why do ArtiG^mCh/PUM^ exhibit a limited size with respect to the protein-based ArtiG^mCh^? Theoretical studies suggest that coarsening by Ostwald ripening could be counterbalanced if non-equilibrium chemical reactions are present in the droplets^65–67^. In *X. laevis* oocytes, nuclear actin network has been shown to stabilize nucleoli against coalescence^68^. Recent work in yeast provides a model to explain the size of P-bodies from the intrinsic interactions of P-body components^69^. In our system, we hypothesize that steric hindrance at the surface of ArtG^mCh/PUM^ might impede growth and coalescence (Fig. 5e). The recruitment of endogenous RNA and associated RBPs at their surface may reduce subsequent sub-unit addition, thus limiting individual ArtiG^mCh/PUM^ growth. In addition, this recruitment possibly introduces enough spacing between nearby ArtiG^mCh/PUM^ to obstruct fusion events. In support of this scheme, our results show that the density of extrinsic interactions that the artificial condensates make with intracellular components scales the size of the final assemblies (Fig. 5a, b). Furthermore, the fusion propensity of ArtiG^mCh/PUM^ can be in part restored in cell lysates by RNA digestion (Supplementary Fig. 7), which we interpret as the reduction of steric hindrance rather than granule aggregation as observed in prion-like proteins^23^. This model in which steric hindrance at the surface is a physical parameter that regulates the morphology of cellular condensates opens the interesting possibility that other biomolecules than RNA/RBPs could modulate the size of RNP granules, provided they share some general features with RNA (e.g., multivalency, promiscuity, and network of interactions).

This work illustrates the interest of developing bottom-up approaches to dissect the general principles of RNP granule formation and function within living cells, taking into account the intracellular complexity of composition and interactions. Our work suggests that RNA and associated proteins can act as molecular cues that not only spatially seed higher-order RNP assemblies, but also specify their ultimate composition. Furthermore, the docking of biochemically heterogeneous phases arises as a critical parameter that affects the size of the condensates by limiting the growth by component addition or coalescence.

## Methods

### Experimental model

Human epithelioid carcinoma HeLa (ATCC, ccl-2) and embryonic kidney HEK-293 (ATCC, CRL-1573) cells were maintained in Dulbecco’s modified Eagle’s medium (with 4.5 g/L d-glucose, HyClone) supplemented with 10% fetal bovine serum (PAA Laboratories) and antibiotics, at 37 °C in a 5% CO_2_ humidified atmosphere. Cells were regularly tested for mycoplasma contamination.

### Plasmids

All the constructs were fused to a C-terminal Myc-tag and sub-cloned into pcDNA 3.1 plasmid (Invitrogen). The sequences of the primer used in this study are listed in Supplementary Table 1.

First, the human FKBP (NM_000801.4) was fused to the N terminus of the light chain of human Ferritin (M11147.1 L), with XhoI and BamHI restriction sites in between, for modular sub-cloning. The FKBP-Ferritin cassette was then inserted between NheI and XbaI restriction sites in pcDNA 3.1 FKBP-mCherry-Ferritin was obtained by inserting mCherry sequence between XhoI and BamHI restriction sites in FKBP-Ferritin construct.

Self-interacting F36M-FKBP (Fm) mutant was generated by point mutation of human FKBP^26^, obtaining FFm construct. Fm-mCherry-Ferritin (mCherry-FFm), Fm-EGFP-Ferritin (EGFP-FFm), and Fm-tGFP-Ferritin (tGFP-FFm) were obtained by inserting the respective sequences between XhoI and BamHI restriction sites in FFm construct.

The PUM-HD (Gly-828 to Gly-1178) of human *PUM1* gene (NM_001020658.1) was amplified by PCR from pEF-BOS_FlagPUM1 plasmid, a gift from T. Fujita^31^. FKBP-PUM.HD-Ferritin and Fm-PUM.HD-Ferritin (PUM.HD-FFm) were obtained by inserting PUM.HD sequence between XhoI and BamHI restriction sites in FKBP-Ferritin and FFm constructs. FKBP-mCherry-PUM.HD-Ferritin was obtained by inserting mCherry sequence between XhoI restriction site in FKBP-PUM.HD-Ferritin. PUMdCt mutant was generated by truncating the C-terminal region of PUM.HD by PCR amplification (Gly-828 to Ala-1080 of human *PUM1* gene) and inserted between XhoI and BamHI restriction sites in FFm construct to obtain Fm-PUMdCT-Ferritin (PUMdCT-FFm).

The plasmid for cytoplasmic poly(A)-binding protein (PABP-EGFP) expression is a gift from M. W. Hentze^70^.

### Transfection

All the constructs were expressed by transient transfection using Lipofectamine 2000 (Invitrogen) according to the manufacturer’s protocol. Unless mentioned, cells were imaged or analyzed 24 h after transfection.

To obtain ArtiG^mCh^, cells were transfected with an equal plasmid ratio (of 1:1 wt/wt) of FFm and mCherry-FFm constructs. To obtain ArtiG^mCh/EGFP^, cells were transfected with an equal plasmid ratio (of 1:1 wt/wt) of mCherry-FFm and EGFP-FFm constructs. To obtain ArtiG^PUM/mCh^, cells were transfected with different plasmid ratios (of 1:1, 5:1, 1:10 wt/wt) of PUM.HD-FFm and mCherry-FFm constructs. Unless mentioned, ArtiG^PUM/mCh^ correspond to a transfection ratio of 5:1. To obtain ArtiG^PUMdCT/mCh^, cells were transfected with an equal plasmid ratio (of 1:1 wt/wt) of PUMdCT-FFm and mCherry-FFm constructs.

### Live-cell imaging

HeLa cells were cultured on 35 mm imaging µ-dishes with polymer coverslip bottom (Ibidi). Live-cell imaging was performed on a Zeiss LSM 710 META laser scanning confocal microscope using an ×63 oil-immersion objective (PlanApochromatic, numerical aperture (NA) 1.4), at 37 °C in a 5% CO_2_ humidified atmosphere. Microscope hardware and image acquisition were controlled with LSM _Software_ Zen 2009. Images were analyzed using ImageJ (NIH) and Icy^71^.

### Transmission electron microscopy

Ultrastructural studies were carried out on glutaraldehyde-fixed cells embedded in Epon™ 812 as described in Souquere et al.^72^. Cells were fixed for 1 h at RT in 1.6% glutaraldehyde (Taab Laboratory Equipment, Reading, UK) in 0.1 M phosphate buffer pH 7.3. Then, cells were pelleted and dehydrated in increasing concentrations of ethanol and embedded in Epon. Polymerization was carried out for 48 h at 64 °C. Ultra-thin sections were collected on Formvar-carbon-coated copper grids (200 mesh).

Anti-Ferritin and anti-DDX6 immunogold labeling was performed on ultra-thin sections of glutaraldehyde-fixed and formaldehyde-fixed Lowicryl K4M-embedded cells, respectively. Embedding was at −20 °C in an AFS2 Freeze Substitution Processor apparatus (Leica Microsystems), polymerization under UV light was for 2 days at −20 °C, followed by 2 days at 20 °C. Electron microscopy grids were incubated with the primary antibody (rabbit anti-ferritin, Abcam 75973; rabbit anti-DDX6, Bethyl Laboratories) and subsequently with a goat anti-rabbit secondary antibody coupled to 10 nm gold particles (BB International).

Sections were analyzed with a FEI Tecnai transmission electron microscope and images were taken with a SIS MegaviewIII CCD camera.

### Fluorescence recovery after photobleaching

FRAP experiments on ArtiG^mCh^ in live HeLa cells were performed at 37 °C in a 5% CO_2_ humidified chamber mounted on a Zeiss LSM 710 META laser scanning confocal microscope using an ×63 (NA 1.4) oil-immersion objective. FRAP experiments and image acquisition were controlled LSM Software Zen 2009. After scanning twice to set the initial level of fluorescence, the photobleaching of an ArtiG^mCh^ with a diameter of 2.5 µm was achieved using a circular region of interest and a 543 nm laser. Fluorescence recovery was monitored by acquiring images (208 × 208 in pixels) every 3 s at a scanning speed of 1.24 µs/pixel for up to 6 min. FRAP data were analyzed with MATLAB (Mathworks) to generate the fitting of the recovery curves and the corresponding graphs. To determine spatiotemporal FRAP patterns, kymographs were generated by measuring fluorescence evolution as a function of time across a line of interest, using Icy software^71^.

### Single RNA molecule detection

Single RNA molecule detection was performed according to the previously described smiFISH method^35^.

The sets of transcript-specific probes were designed using the R script Oligostan^35^ and purchased, together with the secondary Cy3 FLAP probe, from Integrated DNA Technologies. The sequences are listed in Supplementary Table 2. The transcript-specific FLAP-structured duplexes for fluorescent in situ hybridization were obtained by mixing 40 pmol of an equimolar mixture of the transcript-specific probes (with a final concentration of 0.833 μM for individual probes) with 50 pmol of Cy3 FLAP, in NEB3 buffer. The reaction mix was sequentially incubated at three different temperatures (at 85 °C for 3 min, at 65 °C for 3 min, and at 25 °C for 5 min).

HeLa cells grown on glass coverslips were transfected with EGFP-FFm and FFm (in a plasmid ratio of 1:9) or with EGFP-FFm, FFm, and PUM.HD-FFm (respectively in a plasmid ratio of 1:7, 5:1, and 5 wt/wt). Twenty-four hours after, cells were fixed with 4% paraformaldehyde (PFA) for 20 min at RT and permeabilized with 70% ethanol in phosphate buffered saline (PBS) overnight at 4 °C. The cells were then washed in PBS and incubated in 15% formamide freshly made in saline-sodium citrate (SSC) buffer for at least 15 min at RT. To prepare the hybridization solution for two coverslips, 50 µl of Mix 1 (5 μl of 20× SSC buffer, 1.7 μl of 20 μg/μl *Escherichia coli* tRNA, 15 μl of 100% formamide, 2 μl of FLAP-structured duplexes, in water) were added to 50 μl of Mix 2 (1 μl of 20 mg/ml RNAse-free bovine serum albumin (BSA), 1 μl of 200 mM vanadyl ribonucleoside complex, 27 μl of 40% dextran sulfate, in water). In situ hybridization was performed overnight at 37 °C by exposing the cells on the coverslips to 50 μl of Mix 1 + Mix 2 in a humidity chamber (10 cm Petri dish with a 3.5 cm Petri dish containing 15% formamide prepared in SSC buffer). After incubation, the cells were washed twice with freshly prepared 15% formamide in SSC buffer at 37 °C for 30 min and twice with PBS for 5 min. Coverslips were mounted with 5 μl of Vectashield antifade mounting medium with DAPI (Vector Laboratories).

Epifluorescence microscopy was performed on an inverted Zeiss Z1 microscope equipped with a motorized stage using a ×63 (NA 1.32) oil-immersion objective.

### Immunofluorescence

Cells grown on glass coverslips were fixed in 4% PFA for 20 min and permeabilized with 0.1% Triton X-100 (in PBS) for 10 min. Next, cells were successively incubated with the primary antibody and the fluorochrome-conjugated secondary antibody for 1 h, with intermediate PBS washing steps, all steps being performed at RT. Nuclei were visualized with Hoechst stain and slides were mounted in SlowFade reagent (Invitrogen). Primary antibodies (diluted to between 1:500 and 1:1000 in PBS containing 0.1% BSA) were rabbit DDX6 (Novus, 1/1000 dilution), mouse EDC4 (Santa Cruz Biotechnology, 1/1000 dilution), and rabbit ATXN2L (Bethyl, 1/500 dilution). Secondary antibodies conjugated to Cy2 (diluted to 1:1000 in PBS containing 0.1% BSA) were purchased from Jackson ImmunoResearch Laboratories. Image acquisition was performed using confocal microscopy as described above. Images were processed with Icy^71^.

For stress induction, cells were treated with 0.5 mM sodium arsenite (Sigma-Aldrich) for 30 min at 37 °C. Nile Red (Sigma-Aldrich) was used as a lipophilic fluorescent dye, which binds to neutral lipids of hydrophobic intracellular structures such as lipid droplets and lysosomal phospholipid inclusions^73,74^.

### Western blotting

Cells were collected in Triton X-100 RIPA buffer and sonicated. After a denaturing step of 20 min at 95 °C in Laemmli buffer, 10 µg of proteins were resolved in 4–15% polyacrylamide gels (Mini-PROTEAN TGX Stain-Free, Bio-Rad) and transferred to a nitrocellulose membrane (Proten 0.2 µm NC, Amersham). Membranes were blocked in TBST containing 5% (wt/vol) non-fat dry milk for 30 min at RT and incubated with the primary antibody overnight at 4 °C. The day after, membranes were washed in TBST, incubated with horseradish peroxidase-conjugated secondary antibody for 1 h at RT, and washed again. Proteins were detected with the chemiluminescence detection reagent Clarity Max Western ECL Substrate (Bio-Rad) and visualized using the ChemiDoc MP Imaging System (Bio-Rad).

Primary antibodies (diluted to between 1:2000 and 1:5000 in TBST containing 5% milk) were rabbit Myc-Tag (71D10, Cell Signaling Technology), rabbit β-Actin (#4967, Cell Signaling Technology), mouse mCherry (Living Colors, Clontech), and rabbit Pumilio 1 (C2C3, C-term, GENETEX). Horseradish peroxidase-linked secondary antibodies (diluted to 1:5000 in TBST containing 5% milk) were anti-rabbit IgG (#7074, Cell Signaling Technology) and anti-Mouse IgG (H + L) (W4021, Promega).

### Organelle-enriched cell extract preparation

Cells were collected in Triton X-100 RIPA buffer and let on ice for 20 min. To accelerate cell lysis, the cell extract was passed twice through a 25 G syringe. Nuclei were removed by low-speed centrifugation during 5 min. The cytoplasmic extract was treated with 4 U/mL of DNAse (Promega) for 1 h on ice and then centrifuged at high speed for 7 min. The supernatant was discarded and the organelle-enriched pellet was collected.

When required, the organelle-enriched extract was treated or not with 2 µg/mL of RNAse A (Promega) during 2 h on ice and then centrifuged at high speed for 20 min to favor fusion events.

### Image analysis

For the quantitative analysis of mCherry-FFm fluorescence as a function of time, time-lapse live imaging was performed on the confocal microscopy setup described above, after 8–10 h of mCherry-FFm or mCherry-FFm/PUM.HD-FFm transfection by acquiring one image every 2.5 or 4 min for 12 h. For each condition, three independent experiments were performed with three different transfections. The dilute cytosolic mCherry-FFm fluorescence (dilute phase) was determined by averaging the mean intensity (a.u.) of a fixed region of interest that was locally positioned through the time lapse to avoid condensates. These measurements were carried out at the two poles of each cell to assess the robustness of the analysis method. The total cytoplasmic fluorescence was determined by measuring the mean intensity of the whole cytoplasm, including the condensates. Automated intensity computing was done with the software Icy^71^, using the ROI Intensity Evolution plugin, and exported in Excel files to be analyzed.

For the quantitative analysis of ArtiG^mCh^ fusion dynamics, we computed the fusion timescale by quantifying the dynamics of coalescence as a function of time. Upon fusion, ArtiG^mCh^ relax exponentially into a large spherical condensate. The distance between two ArtiG^mCh^ was estimated by measuring the long axis of the two condensates at the onset of the fusion (at the maximum of fluorescence of the mCherry fluorescence channel) and plotted as a function of time. Then the fusion time of different fusion events was plotted as a function of the diameter of the final condensates. The fusion time increases with the condensate diameter and displays roughly a linear tendency as for a simple liquid behavior. The slope approximately scales as ~*l* (*γ*/*η*), where *l* is the condensate diameter, *η* the effective viscosity, and *γ* the surface tension. The linear fit between the length and the fusion time allows one to extract a capillary viscosity of about 1 µm min^−1^. Assuming *γ* ≈ *k*_B_*T*/a^2^ ≈ 10 μN m^−1^ (where *a* is the molecular size of the ferritin), we can thus estimate that ArtiG^mCh^ have an effective viscosity of about 10^3^ Pa.s.

The quantitative analysis of ArtiG size distribution was performed using electron microscopy and fluorescence microscopy images depending on the condition. The size distribution of the ArtiG^mCh/PUM^ obtained with a transfection ratio of 1:1 was obtained by measuring the long axis (µm) of 74 structures from 17 different electron microscopy images (50 undefined structures, with an area < 0.04 µm^2^, were neglected as they could also correspond to tangential cuts), from two independent transfection experiments. The size distribution for the ArtiG^mCh/PUM^ conditions (5:1 and 10:1), the mutant ArtiG^mCh/PUMdCT^, and the ArtiG^mCh^ was carried out on fluorescence microscopy images of cells from three independent transfection experiments for each condition. Epifluorescence microscopy was performed on an inverted Zeiss Z1 microscope equipped with a motorized stage using a ×63 (NA 1.32) oil-immersion objective. About 10 *Z*-sections, distant of 0.26 µm, were acquired per field to scan the complete volume of the cells. Image analysis was performed with the software Icy^71^, using Intensity Projection plugin, to project the maximum intensity of the *Z*-stacks on one single two-dimensional image and then using the Thresholder plugin for automated condensate detection. The measurements of the long axis of the detected condensates (330 to 497 condensates depending on the ArtiG condition) were analyzed with MATLAB (Mathworks).

### Statistical analysis

Data treatment was performed with Excel (Microsoft) and MATLAB (Mathworks). For Fig. 4d, Student’s *t*-test (parametric test to compare two observed means) was performed with XLSTAT. For Fig. 4e and Fig. 5b, Kolmogorov–Smirnov’s test (non-parametric test to compare two distributions) and Student’s *t*-test (parametric test to compare two observed means) were performed with XLSTAT and both tests gave similar *p*-values. For the general interpretation of the *p*-values, NS means there is no significant difference between the two distributions, one star means *p*-value < 0.05, two stars mean *p*-value < 0.01, three stars mean *p*-value < 0.001, and four stars mean *p*-value < 0.0001. Error bars always show the SD. For each condition, at least three independent experiments were done (except for electron microscopy imaging).

### Reporting summary

Further information on research design is available in the Nature Research Reporting Summary linked to this article.

## Supplementary information

Supplementary information

Reporting Summary

Description of Additional Supplementary Files

Supplementary Movie 1

Supplementary Movie 2

Supplementary Movie 3

Source Data

## Data Availability

A reporting summary for this article is available as a Supplementary Information file. Source data are provided as a Source Data file. R script Oligostan, ImageJ, and Icy software, used in this study, are available from open source. All data are available from the corresponding author upon reasonable request.
