# Supplementary material for: RNA is a critical element for the sizing and the composition of phase-separated RNA–protein condensates
Source: Nat Commun. 2019 Jul 19;10:3230. doi: 10.1038/s41467-019-11241-6 (PMC6642089; doi:10.1038/s41467-019-11241-6)
Supplement: Supplementary file 1 — Supplementary information [file 41467_2019_11241_MOESM1_ESM.pdf]

Supplementary Information for

RNA is a critical element for the sizing and the composition of phase-separated RNA-protein condensates

Marina Garcia-Jove Navarro[1], Shunnichi Kashida[1], Racha Chouaib[2, 3], Sylvie Souquere[4], Gerard Pierron[4], Dominique Weil[2], Zoher Gueroui[1]*

1- PASTEUR, Department of Chemistry, École Normale Supérieure, PSL University, Sorbonne Université, CNRS, 75005 Paris, France.
2- Sorbonne Université, CNRS, Institut de Biologie Paris-Seine (IBPS), Laboratoire de Biologie du Développement, F-75005 Paris, France.
3- Current address: School of Arts and Sciences, Lebanese International University (LIU), Beirut, Lebanon. Faculty of Sciences, Lebanese University, Beirut, Lebanon.
4- CNRS UMR-9196, Institut Gustave Roussy, F-94800 Villejuif, France

*Correspondence: zoher.gueroui@ens.fr

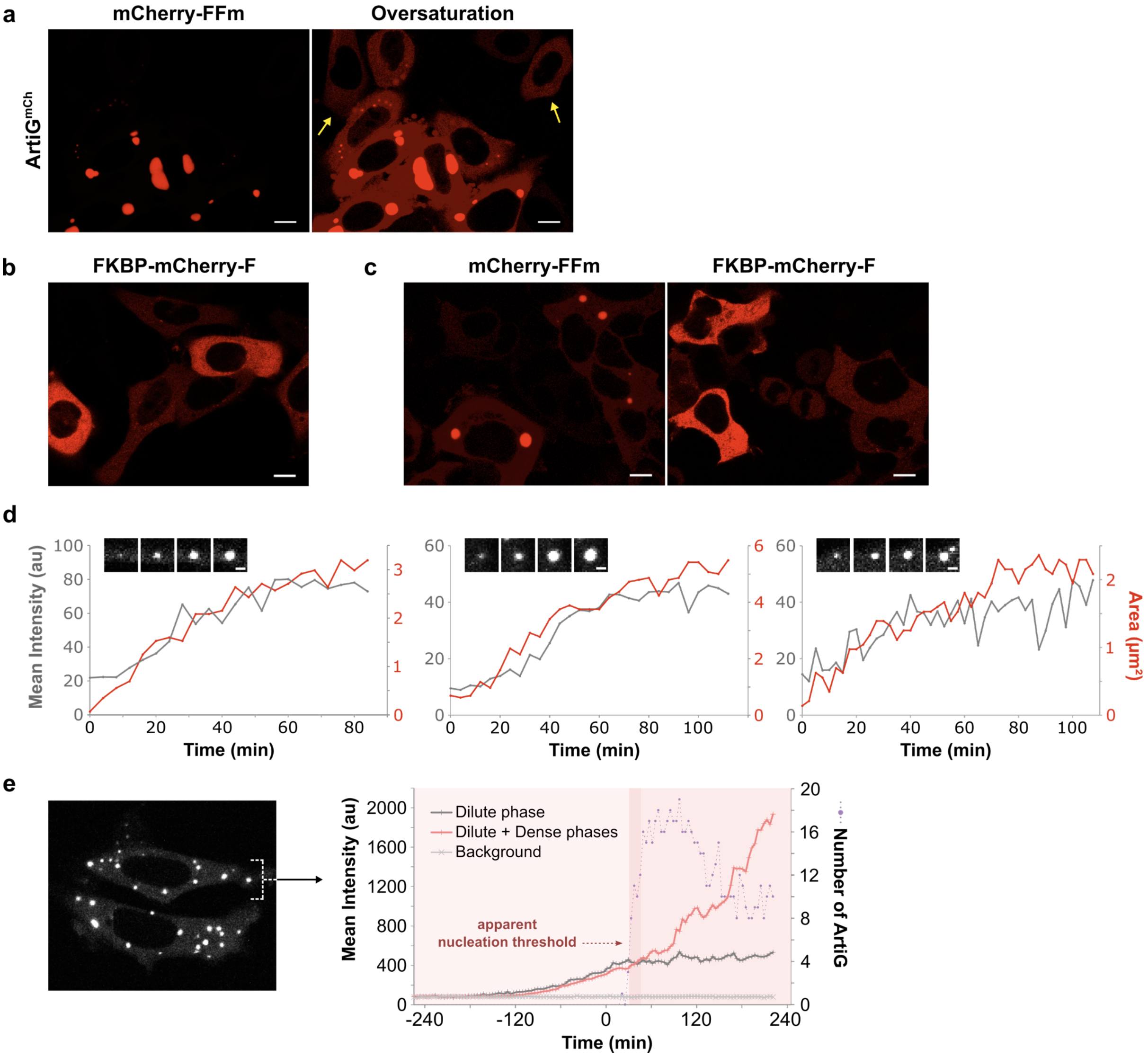

Supplementary Fig. 1 ArtiG are concentration-dependent condensates that form in living cells (**a**) Representative confocal image of HeLa cells exhibiting ArtiG^{mCh}. 24 h after transfection of the multivalent mCherry-FFm construct (red), part of the cells displays fluorescent condensates in their cytoplasm, while others display diffuse fluorescence (yellow arrows). Scale bar, 10 µm. For the purpose of representation, the images in (**a**) and (**c**, left panel) have adjusted contrasts and changed saturation values. (**b**) Representative confocal image of HeLa cells expressing multimeric FKBP-mCherry-ferritin unable to self-interact. Scale bar, 10 µm. (**c**) Representative confocal images of HEK-293 cells 24 h after transfection of mCherry-FFm (red, left panel) and FKBP-mCherry-ferritin constructs (red, right panel). Scale bar, 10 µm. (**d**) Temporal evolution of mean fluorescence intensity (arbitrary unit) and area (µm^2) of individual condensates of three independent experiments and respective time-point images. Scale bar, 2 µm. (**e**) Comparison of the temporal evolution of the fluorescence intensity of the dilute phase, with the total cytoplasmic fluorescence intensity (dilute and dense phases). The violet curve represents the number of granules measured as a function of time. For the purpose of representation, the images in (**c**), (**d**), and (**e**) are slightly saturated.

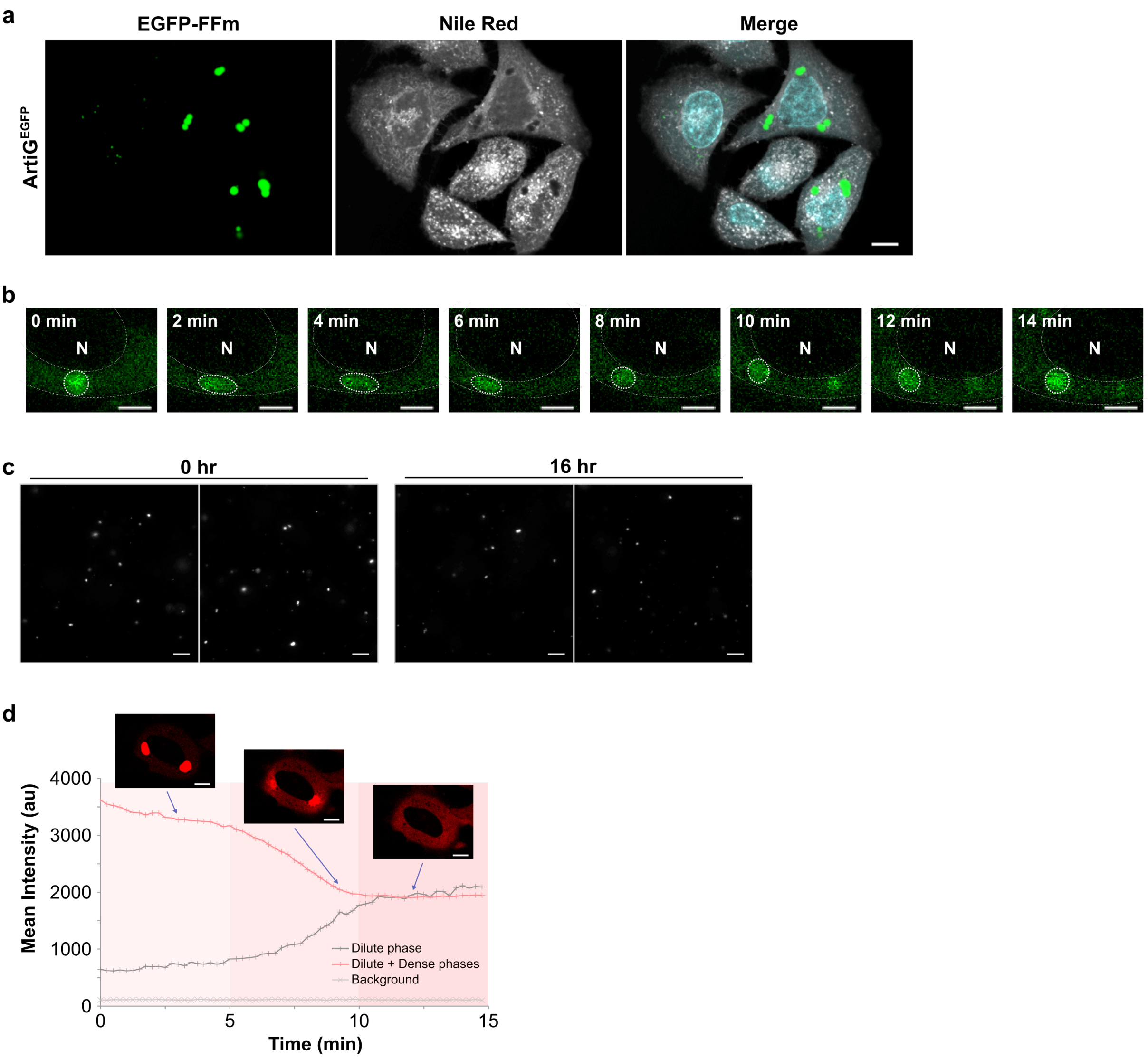

Supplementary Fig. 2 ArtiG are non membrane-bound structures that relax into spherical bodies after mechanical deformation

(**a**) HeLa cells expressing EGFP-FFm construct (green) were fixed and stained with Nile red for intracellular lipid droplets (white) and Hoechst for DNA (blue). Representative confocal image. Scale bar, 10 µm. (**b**). Time-lapse showing the sequence of events of an ArtiG^{tGFP} that deforms under the mechanical action of the nucleus against the plasma membrane and relaxes into a spherical condensate. Single confocal plane of HeLa cells expressing tGFP-FFm construct. N = Nucleus. Scale bar, 2 µm. (**c**) Epifluorescence images of ArtiG^{mCh} observed in cellular lysates after cytoplasmic extract preparation and after an overnight incubation at room temperature. (**d**) Dissolution of ArtiG^{mCh} upon addition of 2,5 µM of FK506 to the culture medium. Comparison of the temporal evolution of the total cytoplasmic fluorescence (dilute and dense phases), with the dilute cytosolic mCherry-FFm fluorescence (dilute phase) as a function of time.

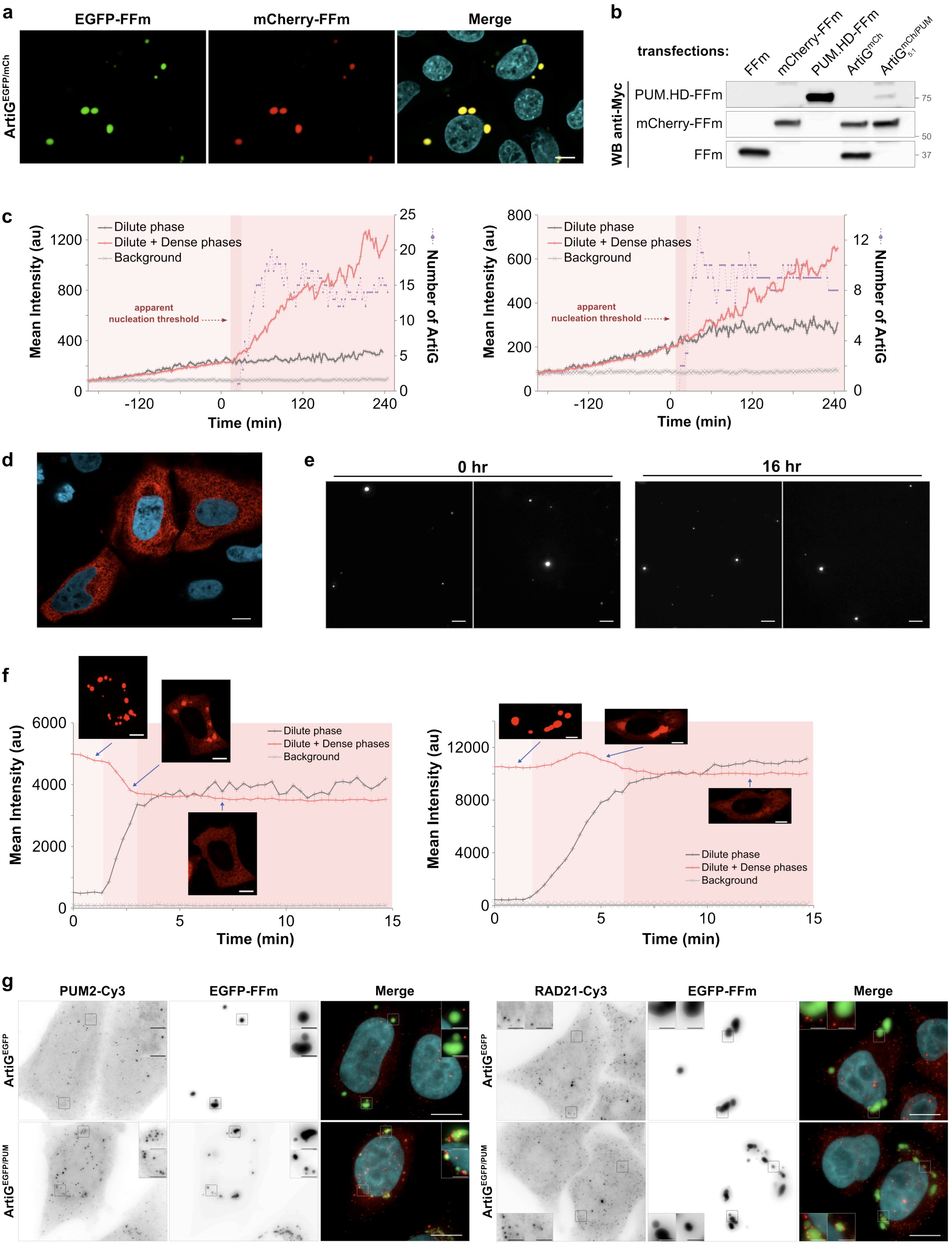

a
EGFP-FFm
mCherry-FFm
Merge
ArtiG^EGFP/mCh
b
transfections:
FFm
mCherry-FFm
PUM.HD-FFm
WB anti-Myc
PUM.HD-FFm
mCherry-FFm
FFm
75
50
37
c
Dilute phase
Dilute + Dense phases
Background
apparent nucleation threshold
Mean Intensity (au)
Number of ArtiG
Time (min)
d
e
0 hr
16 hr
f
Mean Intensity (au)
Time (min)
Dilute phase
Dilute + Dense phases
Background
g
PUM2-Cy3
EGFP-FFm
Merge
RAD21-Cy3

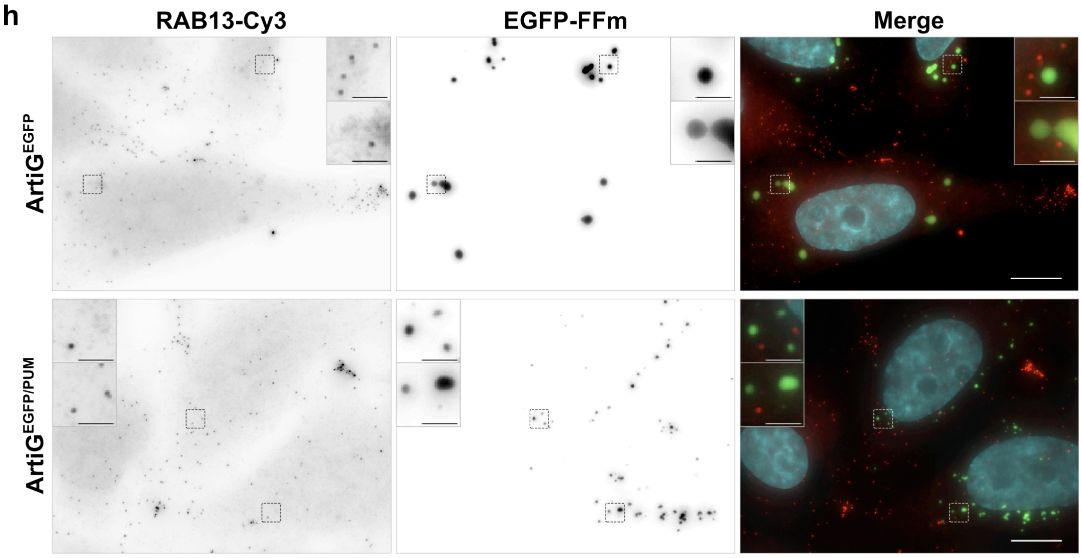

Supplementary Fig. 3 Characterization of ArtiG^{PUM}

(**a**) Representative confocal images of HeLa cells containing ArtiG$^{EGFP/mCh}$ hybrid condensates (yellow in merge), 24 h after transfection of EGFP-FFm (green) and mCherry-FFm (red) constructs. Scale bar, 10 µm. (**b**) Western blot showing expression of the indicated POI-FFm constructs in HeLa cells 24 h after transfection. In Fig. 3 and Supplementary Fig. 3, ArtiG$^{mCh/PUM}$ correspond to the transfection of mCherry-FFm and PUM.HD-FFm constructs in a plasmid ratio of 5:1. Scale, kDa. (**c**) Comparison of the temporal evolution of the dilute phase fluorescence intensity (excluding the fluorescent condensates), with the total cytoplasmic fluorescence intensity (dilute and dense phases) of the cells represented in Fig. 3a. The violet curve represents the number of granules measured as a function of time. (**d**) Representative confocal image of HeLa cells expressing FKBP-mCherry-PUM.HD-ferritin construct (red), which is multimeric but unable of self-interacting. Scale bar, 10 µm. (**e**) Epifluorescence images of ArtiG$^{mCh/PUM}$ observed in cellular lysates after cytoplasmic extract preparation and after an overnight incubation at room temperature. (**f**) Dissolution of ArtiG$^{mCh/PUM}$ upon addition of FK506. Comparison of the temporal evolution of the total cytoplasmic fluorescence (dilute and dense phases), with the dilute cytosolic mCherry-FFm fluorescence (dilute phase) as a function of time. (**g**) Epifluorescence imaging of PUM2 and Rad21 smFISH-Cy3 (red in merge) in HeLa cells expressing ArtiG^{EGFP} (green in merge, upper row) and ArtiG$^{EGFP/PUM}$ (green in merge, lower row). Nuclei were stained with DAPI (blue). Scale bar, 10 µm. Zoom, 2 µm. (**h**) Epifluorescence imaging of RAB13 smFISH-Cy3 (red in merge) in HeLa cells expressing ArtiG^{EGFP} (green in merge, upper row) and ArtiG$^{EGFP/PUM}$ (green in merge, lower row). Nuclei were stained with DAPI (blue). Scale bar, 10 µm. Zoom, 2 µm.

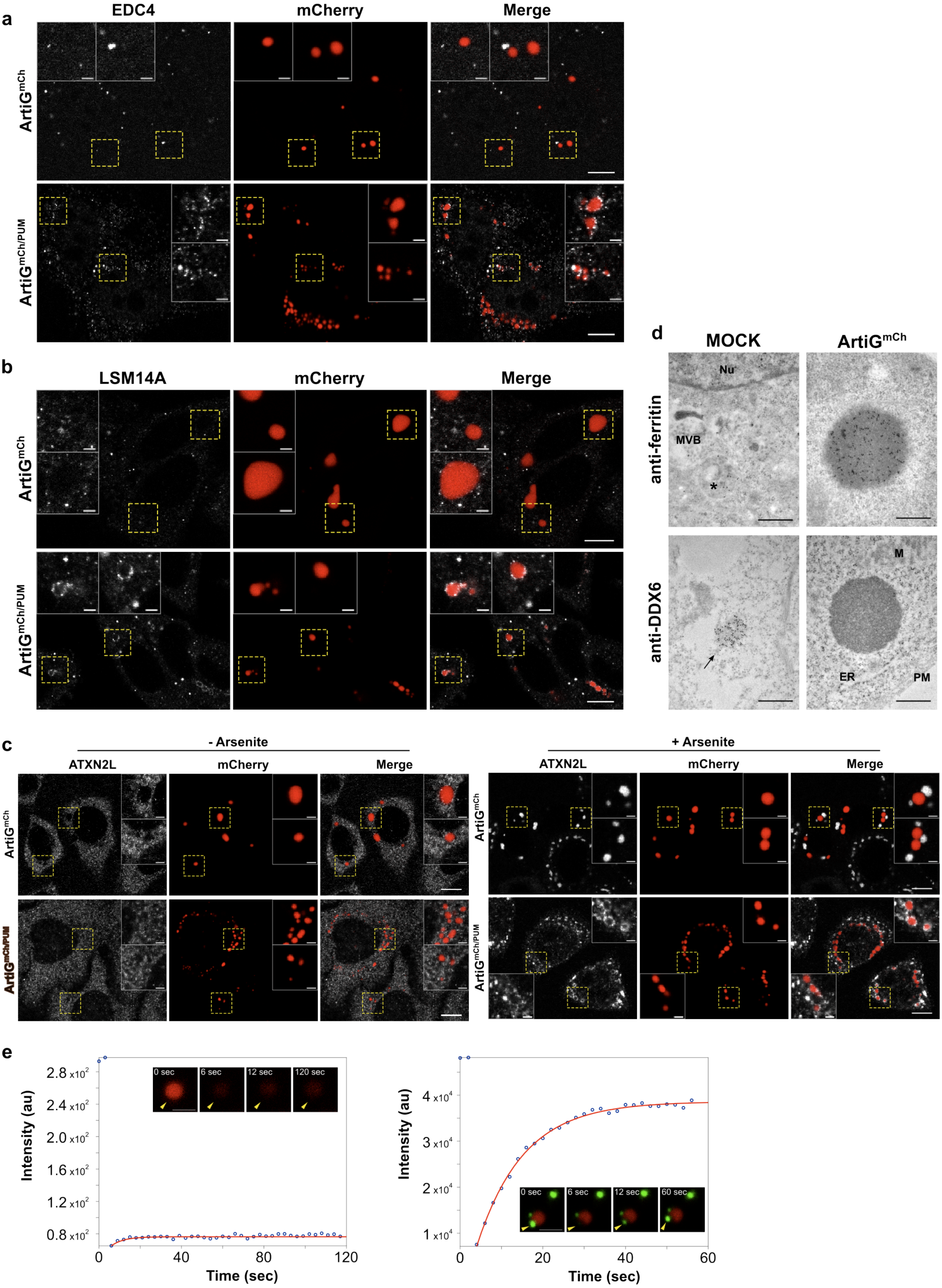

Supplementary Fig. 4 ArtiG^{PUM} are enriched in P-body proteins in non-stressed cells and recruit SG proteins under stress conditions

(**a**) HeLa cells expressing ArtiG^{mCh} and ArtiG$^{mCh/PUM}$ (red) were fixed and analyzed by immunofluorescence using antibodies recognizing endogenous EDC4 (white). Confocal images.

Scale bar, 10 µm. Zoom, 2 µm. (**b**) HeLa cells expressing ArtiG^{mCh} and ArtiG$^{mCh/PUM}$ (red) were fixed and analysed by immunofluorescence using antibodies recognizing endogenous LSM14A (white). Confocal images. Scale bar, 10 µm. Zoom, 2 µm. (**c**) HeLa cells expressing ArtiG^{mCh} and ArtiG$^{mCh/PUM}$ (red) were treated or not during 30 min with arsenite, fixed and analysed by immunofluorescence using antibodies recognizing endogenous ATXN2L (white). Confocal images. Scale bar, 10 µm. (**d**) Immunodection of DDX6 and ferritin in mock and ArtiG^{mCh} expressing cells. P-bodies were heavily labelled by DDX6 in mock-treated cells while ArtiG^{mCh} were not significantly recruiting DDX6. In mock-treated cells, endogenous ferritin was detected at low level in endosomal structures (star) including multi-vesicular bodies (MVB), when ArtiG where heavily labelled upon expression of mCherry-FFm. Nu = Nucleus, M = Mitochondrion, ER = Endoplasmic reticulum, PM = Plasma membrane. Scale bar, 500 nm. (**e**) Examples of recovery of fluorescence intensity after photobleaching of an ArtiG$^{mCh/PUM}$ granule (red, left panel) and of a micrometric patch of EGFP-labelled LSM14A (green, right panel).

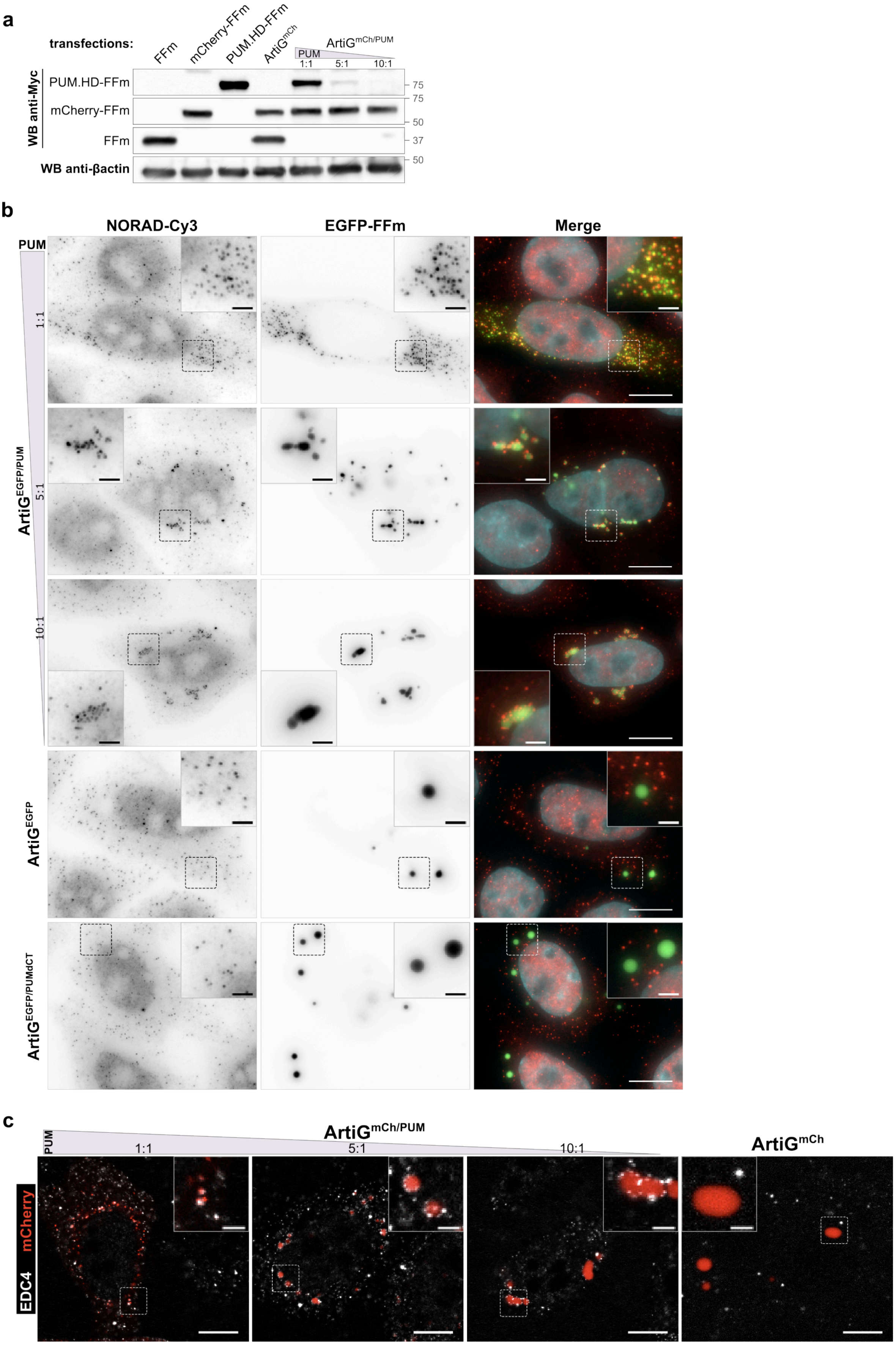

a
transfections:
FFm
mCherry-FFm
PUM.HD-FFm
ArtiGmCh
ArtiGmCh/PUM
PUM
1:1
5:1
10:1
WB anti-Myc
PUM.HD-FFm
mCherry-FFm
FFm
WB anti-βactin
75
75
50
37
50
b
NORAD-Cy3
EGFP-FFm
Merge
PUM
1:1
5:1
10:1
ArtiGEGFP/PUM
ArtiGEGFP
ArtiGEGFP/PUMdCT
c
ArtiGmCh/PUM
PUM
1:1
5:1
10:1
ArtiGmCh
EDC4
mCherry

Supplementary Fig. 5 ArtiG$^{mCh/PUM}$, with different PUM relative ratios, are enriched in RNA and in P-body proteins

(**a**) Western blot showing expression of the indicated FFm constructs in HeLa cells 24 h after transfection. For ArtiG$^{mCh/PUM}$ lanes (from left to right), mCherry-FFm and PUM.HD-FFm constructs were transfected in a plasmid ratio of 1:1, 5:1 and 10:1, as indicated. Scale, kDa. (**b**) Epifluorescence imaging of anti-NORAD lncRNA smFISH-Cy3 (red) in HeLa cells expressing ArtiG$^{EGFP/PUM}$, ArtiG^{EGFP} and ArtiG$^{EGFP/PUMdCT}$ (green). Nuclei were stained with DAPI (blue). Scale bar, 10 µm. Zoom, 2 µm. The panels show the cells enlarged in Fig. 5c. (**c**) HeLa cells expressing ArtiG^{mCh} and ArtiG$^{mCh/PUM}$ (red) were fixed and analyzed by immunofluorescence using antibodies recognizing endogenous EDC4 (white). Confocal images. Scale bar, 10 µm. Zoom, 2 µm.

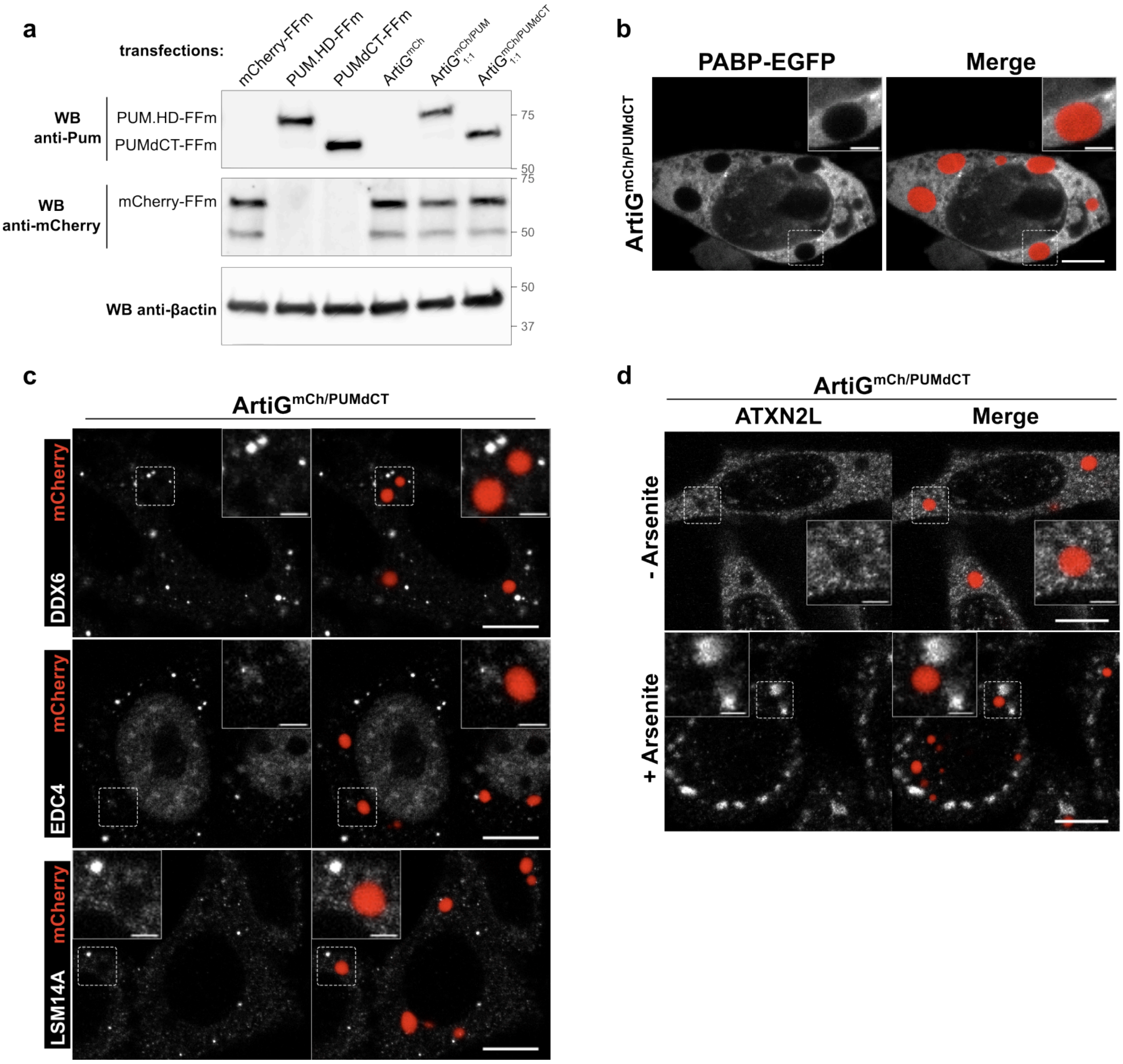

Supplementary Fig. 6 ArtiG$^{mCh/PUMdCT}$ did not display any patchy micrometric assembly of the P-body or SG components, at their periphery

(**a**) Western blot showing expression of the indicated FFm constructs in HeLa cells 24 h after transfection. Scale, kDa. (**b**) To visualize polyadenylated RNAs, a PABP-EGFP fusion (white) was co-transfected with mCherry-FFm and PUMdCT-FFm (red) into HeLa cells 24 h before fixation. Confocal imaging. Scale bar, 10 mm. Zoom, 2 µm. (**c**) HeLa cells expressing ArtiG$^{mCh/PUMdCT}$ (red) were fixed and analysed by immunofluorescence using antibodies recognizing endogenous DDX6, EDC4 and LSM14A (white). Confocal images. Scale bar, 10 µm. Zoom, 2 µm. (**d**) HeLa cells expressing ArtiG$^{mCh/PUMdCT}$ (red) were treated or not during 30 min with arsenite, fixed and analyzed by immunofluorescence using antibodies recognizing endogenous ATXN2L (white). Confocal images. Scale bar, 10 µm. Zoom, 2 µm.

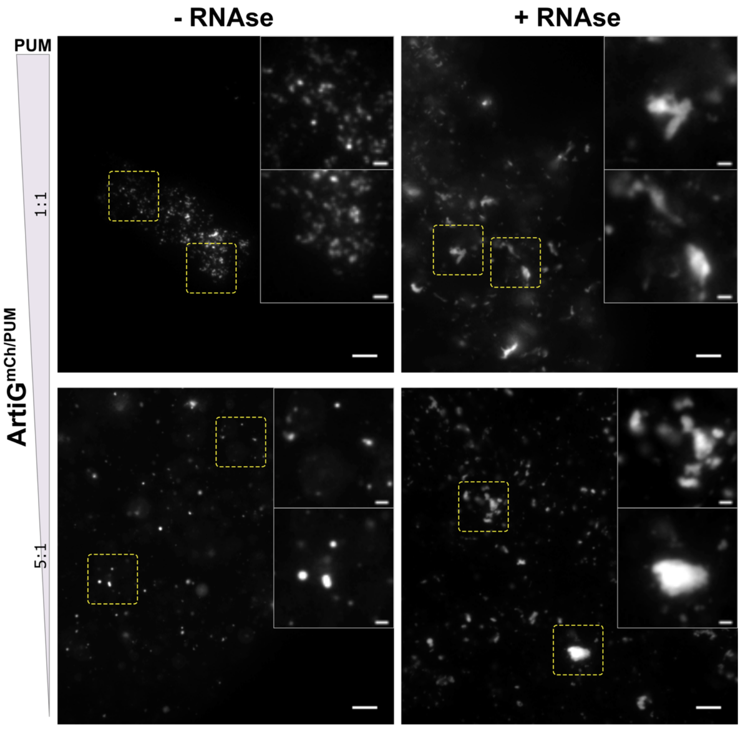

Supplementary Fig. 7 The fusion propensity of ArtiG$^{mCh/PUM}$ can be in part restored in cell lysates by RNA digestion

Epifluorescence imaging of organelle-enriched extracts of HeLa cells expressing ArtiG$^{mCh/PUM}$. Equal amounts of extract were treated or not with RNAse A during 2 h on ice, and then centrifuge at high speed during 20 min to favor fusion events. Scale bar, 10 µm. Zoom, 2 µm.

Construct	ID No.	Oligo name		Sequence
pcDNA 3.1 FKBP-hFerritinL (FT)	SKZ-002	KpnI_NheI_ATG_BamHI_hFtnL_fwd primer	5'-	caaggtaccgctagccaccATGggatccAGCTCCCAGATTCGTCAG
pcDNA 3.1 FKBP-FT	SKZ-003	hFtnL_XbaI_rev primer	5'-	gaatctagaGTCGTGCTTGAGAGTGAgcC
pcDNA 3.1 FKBP-FT	SKZ-006	NheI_FkBP_fwd primer	5'-	caagctagccaccATGGGAGTGCAGGTGGAAACCATC
pcDNA 3.1 FKBP-FT	SKZ-007	FkBP_XhoI_BamHI_rev primer	5'-	cttggatcctgagccctcgagTTCTTCCAGTTTTAGAAGCTCCAC
pcDNA 3.1 FKBP(F36M)-FT	MGZ-049	hFKBP12(F36M)_Fw	5'-	GCTTGAAGATGGAAAGAAAatgGATTCCTCCCGGGACAG
pcDNA 3.1 FKBP(F36M)-FT	MGZ-050	hFKBP12(F36M)_Rv	5'-	CTGTCCCGGGAGGAATCcatTTTCTTTCCATCTTCAAGC
pcDNA 3.1 FKBP(F36M)-mCherry-FT	MGZ-052	XhoI_link_mCherry Fw	5'-	gccgatctcgagggcggtagtggaggcagcGTGAGCAAGGGCGAGGAGGA
pcDNA 3.1 FKBP(F36M)-mCherry-FT	MGZ-053	XbaI_BamHI_link_mCherry Rv	5'-	ggccattctagaggatccagagccgccactaccacccttgtacagctcgtccatgc
pcDNA 3.1 FKBP(F36M)-EGFP-FT	MGZ-085	XhoI link6 EGFP Fw	5'-	GAAGAActcgagggcggtagtggaggcagcATGGTGAGCAAGGGCGAGGA
pcDNA 3.1 FKBP(F36M)-EGFP-FT	MGZ-086	EGFP link6 BamHI Rv	5'-	CTggatccagagccgccactaccaccCTTGTACAGCTCGTCCATGC
pcDNA 3.1 FKBP(F36M)-tGFP-FT	SKZ-008	XhoI_tGFP fwd primer	5'-	gaactcgagGAGAGCGACGAGAGCGG
pcDNA 3.1 FKBP(F36M)-tGFP-FT	SKZ-009	tGFP_BamHI rev primer	5'-	gttggatccTTCTTCACCGGCATCTGCATC
pcDNA 3.1 FKBP(F36M)-PUM.HD-FT	MGZ-064	EcoRI AfeI Pum1-HD G828 Fw	5'-	gccgatGAATTCcaAGCGCTggcaggagcaggcttttgga
pcDNA 3.1 FKBP(F36M)-PUM.HD-FT	MGZ-065	PUM1-HD G1178 AscI SacII Rv	5'-	gtcaggCCGCGGGGCGCGccccctaagtcaacaccgttct
pcDNA 3.1 FKBP(F36M)-PUMdCT-FT	MGZ-108	PUM1-HD dR7R8 AscI SacII Rv	5'-	gtcaggCCGCGGGGCGCGcctgcaaatttgtgctgactca

Supplementary Table 1. Primers used for cloning

RAD21

Probes	Sequence
hRad21_p01	GGCAATTTCTGTTTTCATGATCGGAATACTCA
hRad21_p02	TCTTTTTGTAGCTGAAGGCTATCAGTCATAAC
hRad21_p03	TGTTGTTTCAGATACCATCCCTAAGGAGAGTG
hRad21_p04	AAATCTAAGTTTTCTCAAAGGGTTCTGTGTCC
hRad21_p05	ATATAATATGGAACCTTGGTCCAGGTGTTGC
hRad21_p06	AATTGTCTTGCTATCCAACTCTTTGACACT
hRad21_p07	GCAAATGCTTCTTCCTCATTTGGAACAAGTG
hRad21_p08	AAGCTGAACTGCTGGGCCACATCGAT
hRad21_p09	TTTCCTGTGATAGATTCGAACTACTCCCAG
hRad21_p10	AGAGATGTCCTGATGTCCGTAATGCCATTT
hRad21_p11	CAAACAAGATGGCTGCCGTTACGCCGC
hRad21_p12	AGCTTTGCTCATTTTGGAGCCTCACTAAAAT
hRad21_p13	AATTTCGCGTACACCTCTGCTCATTATGG
hRad21_p14	TGGGGTGCTGTTTTCTCCATCAGAACACA
hRad21_p15	TGATGTCACTGTACGGTTCTTCCTGTGTC
hRad21_p16	CTGTAGAACTTTGCGGCAGCTTGTTTTCTGT
hRad21_p17	TGAAGCATCTGCTGAGTCCTTTTGTTCCA
hRad21_p18	GATGCATCTTCATCCTCTTCCTCTTC
hRad21_p19	CATCTTCTTTTTCCTTCTCTTTCTCCTTCT
hRad21_p20	TTTTCTGGCAGAAGTTCTAACTCTGGTATTAG
hRad21_p21	GACAGATATTTGGAGGTTCTTCTGGGGGAAGC
hRad21_p22	TACAGGTGGTATTTCCATCTGCTCTACC
hRad21_p23	ATTTGTCCAGCTTTTCGCTTAACTCCCT
hRad21_p24	GGTGGTGGTGGAGGCATAGCTGACTCA

PUM2

Probes	Sequence
hPum2_p01	GTCCTAGGTCCGGGCTATTCTTCAAATAATAC
hPum2_p02	TGAGTAACACACTTTTCTACTACATTGCTGGC
hPum2_p03	GCCATACTGATCCTGTACCAACTGCT
hPum2_p04	TGCAATGCTCTAGGATGCGCTGAATTACT
hPum2_p05	AGCACATGACCATCCAGCTCCTTTAC
hPum2_p06	TGCTTTCTGAATAACGCGGCAGCCATAC
hPum2_p07	GGCTGCTGGAGCTAAATAGACTGGAAGTGC
hPum2_p08	CCATGTGATGAAAGTGATGGCGGTGGCGTAAG
hPum2_p09	GCTGCTACTAGATCTTTTGTACAAGTCAGAGC
hPum2_p10	GTAGATAGAGACTCTCTCCTTGTGGCACTA
hPum2_p11	ACTGAGGGCTGAGCCTATAGCAGCAC
hPum2_p12	AATTGGCCGAAACAGACCATTTGTGCTGC
hPum2_p13	TAGGCAGTTGGAGCTAGTACTTGATAGCCTG
hPum2_p14	CCCTGATTGGGAGTAAGAGGACGCTGACC
hPum2_p15	TGCTGCTGAAATAAGTTGGCTGGATACACC
hPum2_p16	AGCCAATCCTGCTGCAGTATACGGATC
hPum2_p17	GCTGAGAATACACCAGCTATATGTGGCTG
hPum2_p18	GTCCATTGGTACCTGATTACCAGGATAGTCA
hPum2_p19	CTGTAAGGATTCCAGACCAACTTGTTCCATGG
hPum2_p20	TGGTTTATTAGCTGTAGGATTAGGAAGAGGCC
hPum2_p21	AGCGCTCAACTACTTCAGTTGGAGAGGCT
hPum2_p22	TCGGCATCCATTCCATTTGGCAAACCT
hPum2_p23	AAACCCTGTCCAGATCTTCTCTGTACCA
hPum2_p24	TTACAGCATTCCATTTGGTGGTCCTCCAAT

NORAD

Probes	Sequence
Norad_p01	AGGATGTCTAGCTCCAAGGGGTGGACTAA
Norad_p02	CTGCAACTTCCGCCTCCCAAGTTCAA
Norad_p03	CGCTGTAAACAGGATGGCATAGAGCTCTC
Norad_p04	TATCGCTTCCAGAGGCCGTCTTAACAAC
Norad_p05	CTCTTTCCATCTAGAAGGGCTAGATGTGAC
Norad_p06	AACGGGCCAAACGTGGCCTGTCATTC
Norad_p07	CACAGCAGAGGCCTCCGTTATCTGCA
Norad_p08	AAGGTCACTCCCAGAGGACAGGCCTT
Norad_p09	GTGGGCACCACCACGACCAGCTAATT
Norad_p10	GATGGTCCTGATCTCTTGACCTCGTGA
Norad_p11	CCACTGCGACAGGCCGTTGTACACTT
Norad_p12	CTCTCCACCACCAACCTGATGGATAT
Norad_p13	GCACGTGTCACTTAGAGCTGATGTTATCTC
Norad_p14	CCCACCTCCCAGGTGGTTCAACAATT
Norad_p15	CCTTCCCATCTCCATCAACCCAGAAG
Norad_p16	GACTAGATGTTGTCAATTAGGACTCGTCTGTC
Norad_p17	TTCTCTTCCTTCTCAGGTCTTCCAGC
Norad_p18	AAGGTTGGGGTGGAGTTGAGAGCAGCTTTTTC
Norad_p19	GTAAATCTTCCCAGAGGGTGGTGGGCATTT
Norad_p20	CCATTTCTCTCTTCCGACAGCAAAGTCTG
Norad_p21	TTGAGTGTCTTCTAAATAGGAACATTCTGGCC
Norad_p22	CTCAGCCTCTCGAGTAACTGGGGCTA
Norad_p23	CCATCTGTAATGCTTAGGGGGGGTTTTAACAA
Norad_p24	CCTTCCTTCTCTGCCTTCAATCCAGT

RAB13

Probes	Sequence
hRAB13_01	CAAGCCCCTCTGCTATTTCTCCCCTGCTCA
hRAB13_02	TCAGTACTGGGAGGCTTGTTGCCGTTTCCTG
hRAB13_03	CTCGAAAGATTTCTCATCCGTGATGTCGTAT
hRAB13_04	AAAGGTGACGAAAATTCTGCATGGAGGCTTA
hRAB13_05	AGCAAATTCCCTAGTGTAGTGCCGAGCTA
hRAB13_06	GAGCCAACTTATCGGCCTGCTCCTTCTGCA
hRAB13_07	GCCTCCATGTCACATTTGTTCCCCAGCAA
hRAB13_08	CTATATCCACAGTGCGGATCTTGAAATCAATTCA
hRAB13_09	AACCAGAATTGTCGTGAGACTCTTCCTGTCT
hRAB13_10	CAGGGAGCACTTGTTGGTGTTCTTCTTGTCACAA
hRAB13_11	GCCAGCCGTGTCCCAGACTTGTAGTTTGATCTT
hRAB13_12	CAGTTATACCAGCTGACAACTCTCTGAATTCAGT
hRAB13_13	TCTTAGAGCCCTCTACTCTTGGTATCCCACC
hRAB13_14	CAATAATCCAAGTTACCCAAAACAGGAACATTTG
hRAB13_15	GCTCCACGGTAGTAGGCAGTAGTTATTGTCTTG
hRAB13_16	ATGTTTGCCCTGAAAACCCAGGTAAGGTC
hRAB13_17	AGGATTACTTTTGAGACAATTAGGAAAGAGGGG
hRAB13_18	ACCCCAGCCGAGGCATTCTCCTTGATGCTTT
hRAB13_19	AACTTGGCACTAGGAAAGTCTGAGGCTA
hRAB13_20	TGGCCTTTAATACCAAGAAAGACCATGACAA
hRAB13_21	TGCCGTTGTCTCCCTCAGGTTCAGCTTC
hRAB13_22	GGAACTAAAAGCCTCATCCACATTCATACTG
hRAB13_23	TCTGTCTCCCCCCAGATATTACTTTCACATTCCT
hRAB13_24	TAGCACTAGTTTCGAAAAATCGGATTCCATG

FLAP X-Cy3

/5Cy3/C ACT GAG TCC AGC TCG AAA CTT AGG AGG/3Cy3Sp/

FLAP Y-Cy3

/5Cy3/AA TGC ATG TCG ACG AGG TCC GAG TGT AA/3Cy3Sp/

Supplementary Table 2. Probes used for smiFISH experiments
