# Supplementary material for: RNA is a critical element for the sizing and the composition of phase-separated RNA–protein condensates
Source: Nat Commun. 2019 Jul 19;10:3230. doi: 10.1038/s41467-019-11241-6 (PMC6642089; doi:10.1038/s41467-019-11241-6)
Supplement: Supplementary file 2 — Reporting Summary [file 41467_2019_11241_MOESM2_ESM.pdf]

nature research

Corresponding author(s): Zoher GUEROUI
zoher.gueroui@ens.fr

Reporting Summary

Nature Research wishes to improve the reproducibility of the work that we publish. This form provides structure for consistency and transparency in reporting. For further information on Nature Research policies, see Authors & Referees and the Editorial Policy Checklist.

Statistical parameters

When statistical analyses are reported, confirm that the following items are present in the relevant location (e.g. figure legend, table legend, main text, or Methods section).

n/a	Confirmed	
☐	☒	The exact sample size (n) for each experimental group/condition, given as a discrete number and unit of measurement
☐	☒	An indication of whether measurements were taken from distinct samples or whether the same sample was measured repeatedly
☐	☒	The statistical test(s) used AND whether they are one- or two-sided<br>*Only common tests should be described solely by name; describe more complex techniques in the Methods section.*
☒	☐	A description of all covariates tested
☒	☐	A description of any assumptions or corrections, such as tests of normality and adjustment for multiple comparisons
☐	☒	A full description of the statistics including central tendency (e.g. means) or other basic estimates (e.g. regression coefficient) AND variation (e.g. standard deviation) or associated estimates of uncertainty (e.g. confidence intervals)
☐	☒	For null hypothesis testing, the test statistic (e.g. F, t, r) with confidence intervals, effect sizes, degrees of freedom and P value noted<br>*Give P values as exact values whenever suitable.*
☒	☐	For Bayesian analysis, information on the choice of priors and Markov chain Monte Carlo settings
☒	☐	For hierarchical and complex designs, identification of the appropriate level for tests and full reporting of outcomes
☒	☐	Estimates of effect sizes (e.g. Cohen's d, Pearson's r), indicating how they were calculated
☐	☒	Clearly defined error bars<br>*State explicitly what error bars represent (e.g. SD, SE, CI)*

Our web collection on statistics for biologists may be useful.

Software and code

Policy information about availability of computer code

Data collection	*Provide a description of all commercial, open source and custom code used to collect the data in this study, specifying the version used OR state that no software was used.*
Data analysis	*Provide a description of all commercial, open source and custom code used to analyse the data in this study, specifying the version used OR state that no software was used.*

For manuscripts utilizing custom algorithms or software that are central to the research but not yet described in published literature, software must be made available to editors/reviewers upon request. We strongly encourage code deposition in a community repository (e.g. GitHub). See the Nature Research guidelines for submitting code & software for further information.

Data

Policy information about availability of data

All manuscripts must include a data availability statement. This statement should provide the following information, where applicable:

- Accession codes, unique identifiers, or web links for publicly available datasets
- A list of figures that have associated raw data
- A description of any restrictions on data availability

The data that support the findings of this study are available from the corresponding author upon reasonable request.

April 2018

Field-specific reporting

Please select the best fit for your research. If you are not sure, read the appropriate sections before making your selection.

☒ Life sciences ☐ Behavioural & social sciences ☐ Ecological, evolutionary & environmental sciences

For a reference copy of the document with all sections, see nature.com/authors/policies/ReportingSummary-flat.pdf

Life sciences study design

All studies must disclose on these points even when the disclosure is negative.

Sample size	For quantitative analysis, we cumulated random images from three independent experiments (different cell transfections), allowing the quantification of the parameters of interest of at least 100 events.
Data exclusions	No data were excluded
Replication	For each condition, at least three independent experiments were done
Randomization	Random acquisition of data and images.
Blinding	n/a

Reporting for specific materials, systems and methods

Materials & experimental systems

n/a	Involved in the study	
☒	☐	Unique biological materials
☐	☒	Antibodies
☐	☒	Eukaryotic cell lines
☒	☐	Palaeontology
☒	☐	Animals and other organisms
☒	☐	Human research participants

Methods

n/a	Involved in the study	
☒	☐	ChIP-seq
☒	☐	Flow cytometry
☒	☐	MRI-based neuroimaging

Antibodies

Antibodies used	Immunofluorescence: Primary antibodies (diluted to between 1:500 and 1:1000 in PBS containing 0.1 % BSA) were rabbit DDX6 (Novus, 1/1000 dilution), mouse EDC4 (Santa Cruz Biotechnology, 1/1000 dilution) and rabbit ATXN2L (Bethyl, 1/500 dilution). Secondary antibodies conjugated to Cy2 (diluted to 1:1000 in PBS containing 0.1 % BSA) were purchased from Jackson ImmunoResearch Laboratories. Secondary antibodies conjugated to Cy2 (diluted to 1:1000 in PBS containing 0.1 % BSA) were purchased from Jackson ImmunoResearch Laboratories.<br><br>Western blot<br>Primary antibodies (diluted to between 1:2000 and 1:5000 in TBST containing 5 % milk) were rabbit Myc-Tag (71D10, Cell Signaling Technology), rabbit β-Actin (#4967, Cell Signaling Technology), mouse mCherry (Living Colors, Clontech) and rabbit Pumilio 1 (C2C3, C-term, GENETEX). HRP-linked secondary antibodies (diluted to 1:5000 in TBST containing 5 % milk) were anti-rabbit IgG (#7074, Cell Signaling Technology) and anti-Mouse IgG (H+L) (W4021, Promega).
Validation	antibodies used were validated in:

Eukaryotic cell lines

Policy information about cell lines

Cell line source(s)	Human epithelioid carcinoma HeLa and embryonic kidney HEK293 cells
Authentication	None of the cell lines used were authentified
Mycoplasma contamination	The cell lines were tested negative for mycoplasma contamination

Commonly misidentified lines
(See ICLAC register)

Name any commonly misidentified cell lines used in the study and provide a rationale for their use.
