# Supplementary material for: RNA is a critical element for the sizing and the composition of phase-separated RNA–protein condensates
Source: Nat Commun. 2019 Jul 19;10:3230. doi: 10.1038/s41467-019-11241-6 (PMC6642089; doi:10.1038/s41467-019-11241-6)
Supplement: Supplementary file 3 — Description of Additional Supplementary Files [file 41467_2019_11241_MOESM3_ESM.docx]

**Description of Additional Supplementary Files**

File Name: Supplementary Movie 1
Description: Dynamics of the ArtiG^mCh^ condensate formation in a concentration-dependent manner within two living HeLa cells

File Name: Supplementary Movie 2
Description: Dynamics of several ArtiG^mCh^ undergoing fusion and relaxing in a single spherical condensate

File Name: Supplementary Movie 3
Description: Dynamics of ArtiG^mCh/PUM^ (obtained in a 10:1 transfection ratio) formation and coalescence
